# Elevating an invisible role: co-designing solutions to optimize medical office assistants in primary care

**DOI:** 10.1186/s12875-025-03155-8

**Published:** 2026-01-06

**Authors:** Jennifer Shuldiner, Apira Ragunathan, Jawairia Mohammed, Amber Khan, Nadine Hare, Tieni Meninato, Jeannie Haggerty, Gary Garber, Robert J. Reid, Meena Andiappan, Danielle Martin, David Kaplan, Tara Kiran, Sylvia J. Hysong, Sabrina T. Wong, Q. Jane Zhao, Carrie Sherlock, France Légaré, Noah Ivers

**Affiliations:** 1 Research and Innovation Institute, Womens College Hospital, Toronto, Canada; 2 Department of Family Medicine, McGill University, Montreal, Canada; 3 Department of Medicine and the School of Epidemiology and Public health, University of Ottawa, Ottawa, Canada; 4 Institute for Better Health, Trillium Health Partners, Mississauga, Canada; 5 DeGroote School of Business, McMaster University, Hamilton, Canada; 6 Department of Family and Community Medicine, University of Toronto,, Toronto, Canada; 7 Ontario Health, Toronto, Canada; 8 Baylor College of Medicine, Michael E. DeBakey VA Medical Center, Houston, USA; 9 UBC Centre for Health Services and Policy Research, School of Nursing, Vancouver, Canada; 10 Institute of Health Policy, Management and Evaluation, University of Toronto, Toronto, Canada; 11 Alberta Medical Association Accelerating Change Transformation Team, Edmonton, Canada; 12 Department of Family Medicine and Emergency Medicine, Université Laval, Quebec, Canada; 13 MAP Centre for Urban Health Solutions, Toronto, Canada; 14 Department of Medicine, University of Toronto, Toronto, Canada; 15 co-being design, Toronto, Canada

**Keywords:** Primary care, Medical office assistants, Health policy

## Abstract

**Background:**

Medical Office Assistants (MOAs) play a vital but often underrecognized role in primary care, managing administrative and clinically-adjacent tasks, allowing the office to run efficiently and effectively. Little is known about how to optimize these team members for the best possible patient and team outcomes. We sought to: (1) explore MOAs’ current roles and experiences; (2) identify barriers and enablers to their work; and (3) co-design scalable solutions to support them in their work.

**Methods:**

This multiprong multi-method study was guided by co-design principles and employed a combination of MOA co-creation workshops, a two-part Delphi survey, and a multi-stakeholder co-creation workshop. MOAs were recruited from a province-wide survey in Ontario, Canada. Four structured workshops with MOAs explored their experiences, challenges, and ideas for improving their work (*N* = 9). We conducted a thematic analysis to identify patterns in participants’ experiences with MOAs. Solutions emerging from the workshops were rated through a two-round Delphi survey with MOAs, clinicians, researchers, and health system leaders (*n* = 32). Solutions were evaluated using the APEASE framework (Acceptability, Practicability, Effectiveness, Affordability, Safety, Equity). Lastly, a multi-stakeholder workshop brought 18 participants together to discuss prioritized solutions and future scaling strategies.

**Results:**

Nine MOAs participated in the co-creation workshops (all female, mean age 43, half identified as racialized). They emphasized their central role in team-based patient care, clinic flow, and health system navigation. Key challenges included a steep learning curve, lack of formal training, frequent workflow changes, emotional strain, and a perception of not being valued by patients or other team members. Staffing shortages and fragmented systems compounded these pressures. The top-rated solutions included creating a professional MOA association, expanding training and safety protocols, defining best practices for MOA roles, and streamlining referrals through centralized systems. Final discussions emphasized the need for leadership engagement, clinic and system-level buy-in, and dedicated funding to support implementation.

**Conclusions:**

The challenges experienced by MOAs may require policy attention to better define and support their role. Targeted investments in training, professionalizing the MOA role, and embedding MOAs more meaningfully in team-based care has the potential to help primary care teams more effectively achieve the quintuple aim.

**Supplementary Information:**

The online version contains supplementary material available at 10.1186/s12875-025-03155-8.

## Introduction

Primary care is the foundation of effective health systems, serving as the first point of contact for patients and ensuring equitable access, continuity, and coordination of care across the health system [1, 2]. The ongoing global health human resources crisis underscores the need for each team member to carry out tasks aligned with their specific training and skills so that primary care teams can function efficiently [3, 4]. Improving efficiency in primary care requires that clinicians work to their full scope in a team-based environment. Medical Office Assistants (MOAs) can help fill gaps by taking on administrative and coordination tasks, allowing clinical staff to focus on their core roles.

Within primary care clinics, MOAs play a pivotal yet often under-recognized role in supporting both patients and clinicians [5, 6]. MOAs typically manage a broad spectrum of administrative duties (e.g., booking appointments, managing referrals, handling paperwork) and in some cases clinically adjacent tasks (e.g., triaging patients, taking vital signs, administering screening questionnaires, pre-ordering standard preventive care tasks, or communicating test results). These responsibilities are essential to clinic operations and care coordination [7].

MOA roles have expanded in response to increasing patient volumes, administrative complexity, and care demands, but they frequently operate in challenging environments with minimal recognition, training, and support [8, 9]. As the first point of contact for patients and key facilitators of communication within and beyond the clinic, MOAs contribute in important ways to patient experience and system navigation. Their close, ongoing interactions with patients afford them deep insight into personal and family contexts, which can be an asset in delivering coordinated, continuous, and patient-centered care. Although substantial research [10] has explored the contributions of physicians, nurses, and allied health professionals, MOAs’ perspectives and experiences remain largely absent from the literature. Addressing this gap is critical, as better supporting MOAs has the potential to improve patient care experiences, and primary care clinic efficiency. This study aims to address this gap by (1) understanding MOAs’ current roles and experiences, (2) identifying the barriers and enablers that affect their work in primary care, and (3) co-creating scalable, innovative solutions that might assist how clinics can more reliably work with MOA’s to implement best practices.

## Methods

### Study design

This multi-method study was guided by co-design principles, with structured co-creation workshops, a two-part Delphi survey, and a multi-stakeholder co-creation workshop. Our approach was guided by human-centred design [11] and co-production principles [12], emphasizing power-sharing, shared decision-making, and iterative development with MOAs as experts in their own experience. We also drew on principles of integrated knowledge translation [13], bringing perspectives of MOAs, primary care clinicians, patient advisors, and healthcare leaders to ensure that the resulting solutions were grounded in the real-world needs and constraints of end-users. Our multi-stage design served distinct purposes: MOA workshops generated experience-based insights and preliminary solution ideas; the Delphi process gathered broader stakeholder input on the feasibility and acceptability of these ideas; and the final workshop refined the top-ranked solutions into actionable steps.

The objective was to engage MOAs in collaboratively identifying their most pressing challenges, and then co-developing scalable, contextually relevant solutions. Co-design is a participatory method that engages stakeholders as equal partners in the design process, recognizing them as experts in their own experiences. As MOAs often come from lower socioeconomic backgrounds and have less institutional power, deliberate efforts are needed to amplify their voices and support their meaningful contribution to primary care. Co-design shifts power, positioning participants as co-decision-makers and researchers as facilitators of a shared process [14]. The study was supported by a project grant from the Canadian Institutes of Health Research. The funder had no role in the design and conduct of the study; collection, management, analysis, or interpretation of the data; preparation, review, or approval of the manuscript; or decision to submit the manuscript for publication. The study was approved by the Women’s College Hospital Research Ethics Board: 2023-0047-E.

### Positionality and reflexivity statement

Our research team included health services researchers, designers, clinicians, and policy advisors, including one team member with previous experience working as a Medical Office Assistant. We acknowledge that our diverse professional backgrounds and varying degrees of institutional power may shape how we interpret MOAs’ experiences. Throughout the study, we engaged in reflexive practice, regularly discussing our assumptions, positionality, and potential biases during team meetings, to ensure that our interpretations remained grounded in participants’ perspectives. The co-design approach, which centered MOAs as knowledge contributors and decision-makers in shaping solution concepts, served as an additional mechanism to enhance the trustworthiness of our analysis.

### Participants and recruitment

MOAs were recruited for the workshops from an online provincial survey conducted by our team at Women’s College Hospital and distributed throughout Ontario primary care clinics. The survey examined MOA roles, responsibilities, work environments, training, and perceived facilitators and barriers. At the end of the survey, respondents were asked if they were interested in supporting the research further by participating in workshops to co-create solutions for improving MOA roles in primary care. Of 939 survey respondents, 62 MOAs expressed interest and were invited; 9 agreed to participate, and 8 completed the full workshop series. We deliberately recruited MOAs from a range of practice settings (e.g., Community health centers and Interprofessional Health Teams), including small, large, and rural primary care clinics. Participants received $50 per hour, and all workshops were held online in the evenings. For the Delphi surveys and multi-stakeholder co-creation workshop, we ensured representation from multiple perspectives by including MOAs, primary care physicians, policymakers, and health system leaders. MOAs were invited from the earlier workshops, while additional participants, including policymakers, were recruited through the research team and project collaborator networks. We used snowball sampling [15] to support the recruitment of a diverse mix across all participant types.

### MOA workshops

#### Data collection

##### Process overview

Workshops began with building rapport and fostering an environment conducive to collaboration, creativity, and shared problem-solving. Virtual workshops were held at times convenient to MOAs to maximize their participation. To ensure an inclusive process, visual and collaborative tools like Miro Board were used, allowing participants to engage with and edit content in real-time. Data were collected throughout the sessions, with participants directly modifying the Miro Board (Miro, https://miro.com), and detailed notes were taken by dedicated note-takers. All workshops were recorded to capture additional insights. Discussion guides can be found in Appendix 1.

##### Workshop 1 – mapping the current state

Aimed to establish an understanding of the current state of MOAs’ work environment and the variability of this experience across Ontario. Participants were invited to share memorable experiences as MOAs, identifying both the challenges and rewarding aspects of their roles. They were encouraged to consider their experiences across multiple levels: the individual (e.g., tasks, roles, and relationships), clinic (e.g., workflows, software, and administrative processes), and system (e.g., policies, regulations, and structural supports). Finally, MOAs were asked to identify opportunity areas and consider ways to improve the MOA experience and primary care for their patients and other team members.

##### Workshop 2 – visioning the ideal day

Participants first engaged in individual reflection before sharing their visions of what their ideal day as an MOA would look like within the group. By stepping outside the limitations of the present, the activity opened space for imagination, supporting a shared vision of the change participants hope to see and clarifying what any solution should strive to enable. From this place, they explored opportunities to enhance the MOA experience, focusing on both personal fulfillment and professional effectiveness, and consider multiple levels: the individual, the clinic, and the system.

##### Workshops 3 and 4 – co-creating solutions

Building on the insights from previous workshops, MOAs prioritized key challenges for which they co-created solutions. Using a structured process, they began by mapping out the root causes of these issues. Next, they developed solutions, with facilitators encouraging consideration of necessary supports and resources, integration of new technologies, establishment of key partnerships or relationships, development of communication processes, and successful implementation.

### Analysis

Multiple data sources were used in the analysis. The Miro board, which captured participants’ real-time contributions, served as the primary analytic dataset. Audio recordings and facilitator notes were reviewed to ensure completeness and accuracy of the Miro board. Using these materials, the research team (J.S., A.R., J.M., A.K., N.H., and T.M.) conducted an inductive reflexive thematic analysis approach, following Braun and Clarke’s methodology, which emphasizes iterative, interpretive theme development inductive thematic analysis [16]. The analytic process included: (1) reviewing all data sources; (2) collaboratively coding and clustering ideas in the Miro board; (3) using affinity mapping to visually group related concepts; and (4) iteratively refining these clusters into themes across several team meetings.

Affinity mapping [17, 18] is a collaborative visual method that enables grouping of related ideas into clusters, groups of thematically linked insights. This approach facilitated the inductive development of themes by allowing the research team to iteratively group, review, and refine data across multiple rounds, helping to surface key challenges and their underlying causes. Affinity mapping complemented the thematic analysis by structuring the data in a way that supported pattern recognition and theme development. Clusters served as the foundation for defining themes and identifying relationships among the data.

To contextualize the findings within a systems perspective, we applied the Iceberg Model [19], which categorizes observable challenges (“events”) and traces their underlying causes through two levels: systemic structures and deeper mental models. This framework enabled us to map MOA experiences from immediate, surface-level issues to clinic- and system-level dynamics, ultimately uncovering the underlying beliefs, assumptions, and cultural norms that perpetuate these challenges. The mental models were co-developed with MOAs in the workshops. We assessed saturation by examining whether themes consistently recurred across participants and across the three MOA groups. Only themes that appeared repeatedly and coherently across groups were interpreted as reaching saturation.

We enhanced credibility through sustained engagement with MOAs across a four-part workshop series, enabling iterative exploration of their experiences. Transferability was supported through purposive recruitment from diverse primary care settings. Dependability was strengthened by clearly documenting study procedures and maintaining an audit trail of analytic decisions, including how workshop activities, affinity mapping, and thematic analysis informed theme development. To ensure confirmability, we triangulated multiple data sources and used a collaborative, multidisciplinary analytic process to ground interpretations in participant perspectives.

### Delphi

#### Data collection

A two-part Delphi survey [20] was designed to evaluate each proposed solution. Participants included MOAs, primary care physicians, policymakers, and health system leaders. The survey was developed specifically for this study, and the final instrument is provided as Supplementary File 1. Round 1: Participants were presented with proposed interventions from the workshops and were asked to rate the solutions using a 5-point Likert-scale (from ‘strongly disagree’ to ‘strongly agree’) on acceptability, practicality, effectiveness, affordability, side-effects, and equity, also known as ‘APEASE’ [21]. Open-ended text boxes captured additional qualitative feedback and insights. A two-week deadline was given to complete the survey, with a reminder email sent after 10 days.

In Round 2, participants reviewed the solutions along with their APEASE scores, viewing them in the order of their preliminary rankings. They were then asked to re-rank them based on feasibility (acceptability, affordability, practicality), effectiveness, equity, and side-effects.

#### Analysis

Each proposed solution was independently scored on a 5-point Likert scale (1 = very low, 5 = very high) for each criterion (i.e., Acceptability, Practicability, Effectiveness, Affordability, Side-effects, and Equity) by members of the project team. Scores were also organized by respondent type. We visualized the highest scoring solution’s profile using a radar (spider) chart. Each axis on the chart represents one of the six APPEASE criteria. Scores were plotted on each axis, and the points connected to form a closed polygon for each solution. The maximum value on all axes was set to 5, in accordance with the Likert scale used. This visual representation allowed for a holistic comparison across the six criteria. To quantify the overall strength of each solution across the APPEASE criteria, we calculated the surface area enclosed by each solution’s radar chart polygon.

In Round 2, data were organized to assess changes in prioritization. We analyzed how each participant ranked the interventions and calculated average rankings to identify the most and least favorably rated solutions by feasibility (acceptability, affordability, practicality), effectiveness, equity, and side-effects, and overall.

### Multi-stakeholder co-creation workshop

#### Data collection

We conducted a final two-hour multi-stakeholder co-creation workshop virtually, bringing a wide range of stakeholders (MOAs, primary care providers, researchers, and health system leaders) to discuss four prioritized solutions identified through the Delphi survey. Participants were divided into four groups to refine the proposed idea, considering its impact and rationale for prioritization. They were then asked to identify enhancements to increase its effectiveness. Finally, using a template, they outlined short-term (e.g., stakeholder mapping and engagement), medium-term (e.g., capacity building, piloting, governance), and one-year (e.g., rollout and evaluation) implementation plans, while also considering potential barriers.

#### Analysis

Workshop notes, recordings, and Miro board notes were organized and synthesized by the research team. The analysis process involved several stages. First, all data sources were compiled and organized by activity and discussion topic. The team conducted an inductive content analysis, clustering similar ideas, identifying patterns, and grouping related concepts into thematic categories. These clusters were iteratively refined to reflect the nuances of participant feedback, and draft summaries were reviewed collaboratively by team members to ensure consistency and accuracy. The final synthesized findings were used to refine and expand proposed solutions and strategies, ensuring they reflected the input, lived experience, and expertise of MOAs, clinicians, and system-level stakeholders. These findings informed the design of actionable next steps for future implementation.

## Results

A total of 12 workshops were conducted, with three groups of three MOAs each participating in a four-part series over the course of four months. All MOAs participated were female with a mean age 43.3 (SD = 6.8); 5 identified as White, 2 as Black, and 2 as East Asian. They worked in Family Health Teams (3), solo practices (2), Community Health Centers (2), and Family Health Organizations (2). Average MOA experience was 10 years (SD = 5.4), with 4 MOAs per clinic on average (SD = 1.8). MOAs who participated in the workshop were similar to overall survey respondents regarding gender (100% vs. 90%), age (43 years vs. 42 years), type of clinic (Type of clinic: Family Health Team: 38% vs. 37%), and years worked in their current clinic (8 years vs. 10 years).

### The current state

**Fig. 1:**
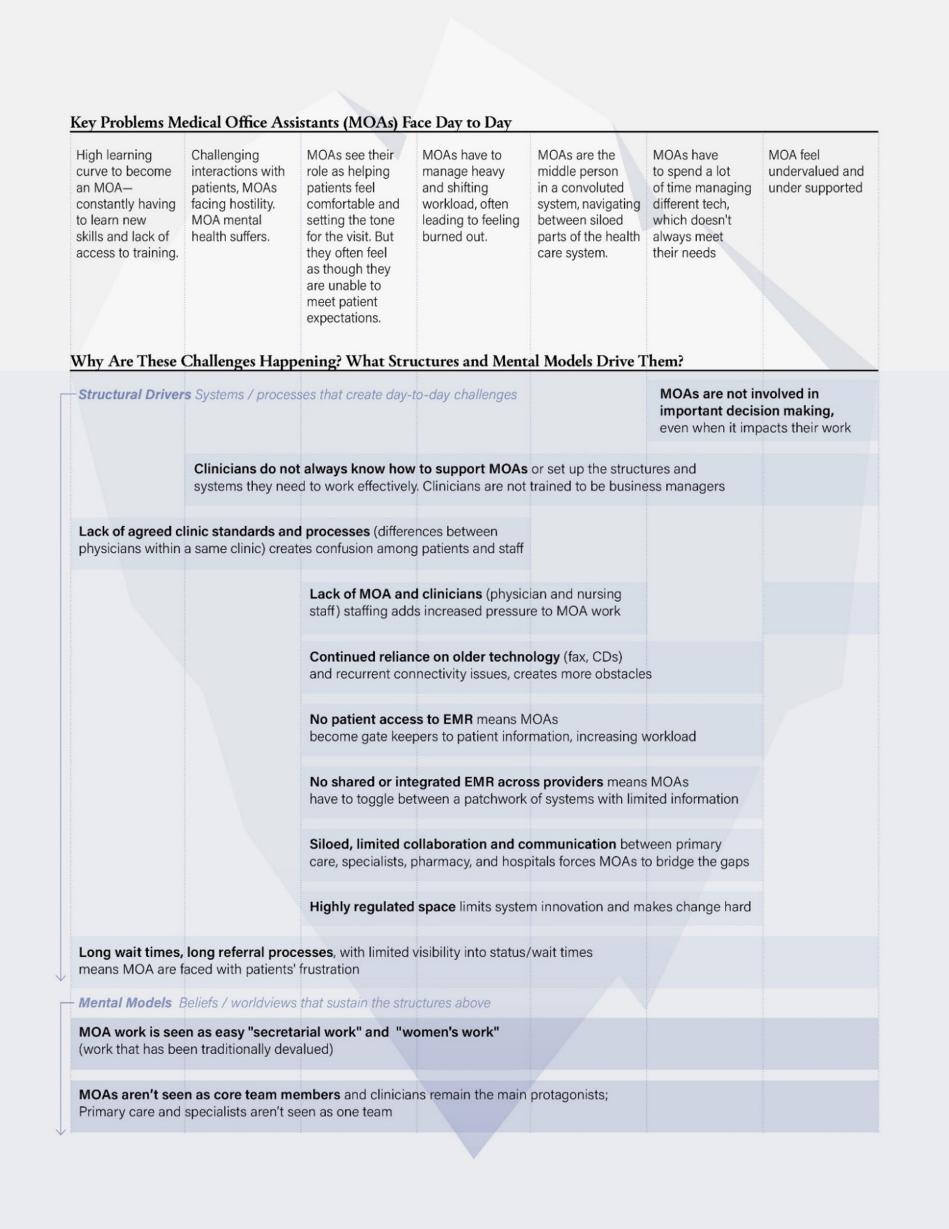
Layers of Medical Office Assistant in Primary Care Experience: An Iceberg Model Analysis

The results are organized using the Iceberg Model (Fig. 1). MOAs described and perceived that they play a central role in primary care, supporting patients, managing clinic operations, and navigating the broader health system. MOAs shared that they face a wide range of interrelated challenges that impact their ability to do their work effectively and sustainably (Table 1). Many MOAs expressed a steep and ongoing learning curve, with new skills often acquired informally due to a lack of structured training and accessible resources. It appears that this is compounded by frequent changes in workflow and technology, as MOAs are expected to quickly adapt to new systems that are not always designed with their needs in mind.

**Table 1:** Medical office assistants’ identified challenges and supporting quotes

Identified Challenges	Supporting quotes
High learning curve to become an MOA - constantly having to learn new skills and lack of access to training.	“Some schools offer EMR training, but only for a specific EMR - leads to learning curves and burden of training costs on small clinics, as well as MOAs leaving” - MOA 1
Challenging interactions with patients, MOAs facing hostility. MOA mental health suffers.	“Patients say “I pay your salary"”… “I am not your secretary” - MOA 7 “We serve in this industry, but we are not servants” - MOA 5
MOAs see their role as helping patients feel comfortable and setting the tone for the visit. But they often feel as though they are unable to meet patient expectations.	“We have so much more on our plate with side tasks that need to be complete; takes a lot of time away from patients” - MOA 3 “We feel like we are just pushing them [patients] through instead of giving them the attention they need or deserve.”- MOA 8
MOAs have to manage heavy and shifting workload, often leading to feeling burned out.	“I have 3 people on hold all the time, plus voicemails every hour - doctors didn’t think about the demand that it [more patients] puts on my role” - MOA 8 “MOAs come and run the clinics for doctors who don’t have much experience running a business/office - doctors have to run businesses despite being trained in medicine.” - MOA 4 “Affects my mental health when patients get mad at MOAs for their lack of time management skills and planning” – MOA 6
MOAs are the middle person in a convoluted system, navigating between siloed parts of the health care system.	“Patients are waiting for referral answers, but we do not know if the referral even went through or not. Sometimes we are tracking referrals for months!” - MOA 1
MOAs spend a lot of time managing different tech, which doesn’t always meet their needs	“Combination of sheer volume and also the process. Also, there is no tracking, office says they sent something, but then we did not receive it because it was sent by fax.” - MOA 3
MOA feel undervalued and under supported	“Sometimes I even downplay myself -I only answer phones – I’m just a receptionist- you feel like the bottom tier people in the health care system - it doesn’t matter that we are educated” - MOA 2

A recurring theme described by MOAs was the emotional and psychological toll of the role. MOAs voiced that they often encounter hostile or frustrated patients, and despite their efforts to create a welcoming environment and set the tone for patient visits, they feel unable to consistently meet expectations, especially given limited time, and systemic constraints.

Operationally, MOAs shared they manage a heavy and constantly shifting workload, frequently serving multiple physicians and taking on administrative tasks without adequate time or support both in small and larger practice settings. They describe that they often act as intermediaries in a fragmented healthcare system, responsible for bridging gaps between primary care, specialists, pharmacies, and hospitals. This includes navigating inefficient referral processes, outdated communication tools, and disjointed EMR systems, all of which consume significant time and energy. It was expressed that these pressures are further intensified by staffing shortages (e.g., nurses, MOAs, physicians), high patient volumes, and a lack of autonomy or involvement in decision-making processes at the clinic level.

### Societal mental models of MOA contributions

Several underlying mental models seem to contribute to the structural and systemic challenges faced by MOAs. First, MOA work is often perceived as clerical “secretarial” or “women’s work”, this is a reflection of gendered and outdated societal norms that undervalue administrative and relational labor. This perception may diminish recognition of the complexity and significance of MOAs’ responsibilities, contributing to insufficient training, low compensation, and limited opportunities for advancement.

Second, clinicians (primarily family physicians) were perceived to be frequently positioned as the primary actors in healthcare, while MOAs appear to be infrequently recognized as core members of the primary care team by physicians and patients. This marginalization limits their involvement in decision-making and reinforces a hierarchical culture that overlooks their contributions to both patient care and clinic efficiency.

Third, the healthcare system seems to operate with a fragmented view of care, where primary care and specialist providers are not perceived as part of the same collaborative team. This fragmentation further isolates MOAs, who are left to navigate disjointed systems and coordinate across silos without sufficient support.

### Co-created solutions

MOAs developed a set of solutions aimed at addressing the structural, interpersonal, and systemic challenges they face (Table 2). The full details of these solutions can be found in Table 3.

**Table 2:** Solutions created by medical office assistants

Solutions Title	Description
Population-wide solutions	
Centralized Referral System for Specialists: Seamless specialist referral and simplified care coordination	A platform designed to centralize all referral requests, tracking and communication. This includes a comprehensive directory of specialists, facilitating direct scheduling with the specialist’s office, and providing clear oversight of referral pathways.
Province-wide Health-care System Education Campaign: Empowering patients to navigate the system and lower the burden on MOAs	This campaign will help the public navigate a complex health system, with topics like where to get help from, making the most of physician visits, patient rights, roles and responsibilities, how to access community and social supports and how to use digital tools and other resources.
Profession-Specific Initiatives	
Professional Association of MOAs: Supporting, connecting, and advocating for MOAs	This association would advocate for the recognition of MOAs, educate the public on the pivotal role of MOAs, act as a resource as well as provide professional development opportunities, and educate care teams on standards.
Maximizing Usage of EMRs and Digital Tools: Training, support, and incentives for full adoption	This initiative will involve comprehensive training for doctors and MOAs on the platforms used in their clinics, as well as financial incentives and funding to support adoption of these tools to their full capacity in clinics.
Clinic-Level Initiatives	
Training and procedures to support safety and enabling caring relationships between MOAs and Patients	Training would be encouraged, subsidized and incentivized. Training would include how to deal with harassment, diffuse situations, and building compassion towards patients. Additionally, managers and family physicians will be encouraged to participate in training on how to support MOAs faced with harassment, and implementing clear procedures in the office on how to respond to such challenges.
Patient Education Initiative: Educating patients on clinic process, roles, and expectations to lower MOA burden and build shared understanding	This initiative will include teaching patients on the roles and expectations of MOAs, standard times for referrals and other health results, outlining and explaining what is billable under OHIP (Ontario Health Insurance Plan), how to use digital programs, and patient rights, roles and responsibilities.
Best Practices for Clinics: Helping physicians manage their clinic and make the most of MOAs	Creating a collection of best practices of clinics, including efficient use of technology, effective communication, patient flow management, time management and workflows, and ongoing training,
Patient Navigation Specialist: A new role to simplify healthcare access	This role would be available for face-to-face support, and act as a point of contact for patients needing extra assistance, helping patients with booking appointments and tests, managing referrals, and connecting them with community resources.

**Table 3:** Detailed solutions created by medical office assistants

Maximizing the EMR and digital tools: Training, Support, and Incentives for Full Adoption		
Problem	Solution	How
• Clinics don’t always use digital platforms to their full potential • This leads to frustration among MOAs and increased workload • MOAs have to negotiate between different physicians’ preferences to use or not use certain functions	Implement training and financial incentives for full adoption of digital tools (such as Ocean, EMRs, etc.): • Provide comprehensive training for both doctors and MOAs on the platforms and all of their functions. • Introduce financial incentives and funding to support clinics and providers in adopting and using digital tools to their full capacity. • Ensure there are clinic processes in place to enable this. For example, ensuring that email addresses are consistently collected and updated for all patients. • Possibility to collaborate with digital tool vendors to improve functionality and better integrate the tool within clinic processes	• Secure financial support for clinics to cover the costs of implementation, training, and ongoing usage of digital tools • Develop and deliver training programmes to ensure proficiency with tools. • Collaborate with digital tool vendors to improve functionality • Collaborate with and support existing local initiatives focused on this (e.g., in Ontario—OntarioMD)

Centralized referral system for specialists: Seamless specialist referral and simplified care coordination		
Problem	Solution	How
MOAs find the referral process time consuming and hard to navigate. This leads to patients being frustrated and MOAs feeling overwhelmed: • Specialists currently accept referrals differently • Lack of transparency who is accepting referrals, for what problems, wait times Lack of transparency and communication regarding where patient is in the referral process (i.e., triage, booked, a consultation note ready)	A streamlined platform designed to manage and coordinate patient referrals from primary care providers to specialists. Instead of relying on fragmented processes—such as phone calls, faxes, or paper forms—the system centralizes all referral requests, tracking, and communication within a single digital interface. The platform would include: • A comprehensive directory of specialists, including their areas of expertise, availability, wait times, can help primary care providers make informed referral decisions. • Facilitate direct scheduling with the specialist’s office, allowing patients to book appointments without back-and-forth coordination. • All referrals would go through this system Provides clear oversight of referral pathways, preventing missed or lost referrals.	• The Ontario Ministry of Health would need to endorse the initiative and possibly mandate the adoption of the system. • Resources to develop, implement, and maintain the centralized referral system. • Specialist and primary care providers to be involved early to ensure their needs are met. • Collaborate with tech companies specializing in healthcare IT

Patient Navigation Specialist: A new role to simplify healthcare access		
Problem	Solution	How
• Patients often struggle to navigate the healthcare system. • MOAs want to support patients, but don’t have the time and capacity to do so • Older patients usually need more support, especially for complex appointments where they have to see multiple specialists. Language barriers can make navigation even harder for patients	Positioned in the clinic, the Patient Navigation Specialist is available for face-to-face support. This role is critical in big clinics where there are multiple doctors and a lot of patients. This could be a new role or MOAs could establish a rotation where one of them takes on that role for the day. They can assist with: • Tech support • Help patients book their own appointments • Manage onboarding processes, ensure all necessary forms are completed accurately Act as a point of contact for patients, addressing questions and concerns	• MOAs would need to be relieved of certain administrative duties or this would have to be a standalone role • Physicians and management would need to create this new role, along with MOA expertise. • Could be piloted with additional government funding • Would require a training program for the navigation specialist • Would require educating patients on the availability of this support role

Health-care System Navigation Education: Empowering Ontarians to navigate the system and lower the burden on MOAs		
Problem	Solution	How
• Many people are unaware of how to navigate health care system • The burden falls on MOAs to navigate the system on their behalf • There is an expectation that MOAs should complete tasks for patients, who have capacity to do so themselves - especially found amongst the younger generation e.g. appointment reminders	Training the general public on navigating the health care system. This can be offered in high school (similar to financial literacy classes), as well as to newcomers who have not experienced the Ontario health care system yet. This could be done through a series of classes, an awareness campaign, or hosted on a portal. Educational material for the general public can also be created and shared on common tasks such as booking appointments. This educational initiative could include: • Understanding Ontario’s Healthcare System • How to access care at the right place and the right time • Understanding appointments and wait times • Making the most of your visit with your provider • Patient rights • Patient roles and responsibilities in managing their health (versus what they can expect from the clinic) • How to access community and social support services How to use digital tools and resources (e.g. My Chart)	• Provincial Lead: to support development of educational materials and promotion to public • Educational materials should be co-created with key stakeholders, including physicians, MOAs, and clinic managers

Clinic Clarity: Educating patients on clinic process, roles, and expectations to lower MOA burden and build shared understanding		
Problem	Solution	How
• Patient expectations for appointments and turnaround times do not align with how the clinic/system works • Lack of understanding among patients on how HC systems works and typical referrals times • Increased burden on MOAs to manage expectations • Physicians don’t always provide consistent information to patients, leaving MOAs to educate (ex: costs of forms, turnaround times for results or appointments)	Clinic-based educational initiative to help patients understand clinic processes, roles, and expectations. This could be shared with patients when being onboarded but would also need to be shared as regular reminders to patients (e.g., in the waiting room, on screens, online portals, etc.). This could include: • Information on MOA roles and expectations • Standard times for referrals, forms, tests, prescription renewal • Outlining of what is billable and what is not under OHIP • How to use digital programmes (e.g., Ocean) • Patient roles and responsibilities • Explaining how billing works in the clinic to build empathy for physicians and MOAs	• Support from clinic management and lead physicians • Resource assessment to evaluate budget for materials and staff time • Identify staff members who can champion the initiative • Provincial bodies could create a template for clinics that could be easily customizable • Engage and co-create with patients, MOAs, and physicians

MOAs are ESSENTIAL to primary care teams: Best practices on making the most out of MOAs in primary care clinics		
Problem	Solution	How
• Doctors are not trained to run a clinic/business, leaving MOAs to figure it out themselves. • Physicians often focus on medical care, leaving MOAs to manage the running of the clinic, with little to no time/resources (e.g. technological tools, referral process, communication within the clinic team) • MOAs have to go looking for resources and there is no one to guide them	Creating a collection of best practices for clinics, to ensure they are making the most out of their MOAs and creating efficient clinic workflows. This document could include: • Efficient use of technology (e.g., automated systems for appointment booking, patient reminders, and follow-ups and Electronic Health Records Optimization) • Effective Communication (e.g., regular meetings, efficient communication channels and feedback mechanisms) • Recommendations for patient flow management (e.g., triage system, check in and check out procedures) • Time Management and Workflow Automation (e.g., time blocking and batch processing) • Ongoing training and development recommendations	• Resource to be co-created with MOAs, patients, allied health and clinicians from various types of clinics throughout the provinces • Support from key provincial associations • Encouragement and incentives to engage and implement the resource

Fostering care between MOAs and Patients: Training and procedures to support safety and enabling caring relationships		
Problem	Solution	How
• MOAs feel that they receive the brunt of patients’ frustration with the system and that some patients treat them poorly because of their status within the clinic. MOAs mental health is severely impacted. • MOAs can feel ill-equipped to address these interactions, clinics don’t always have an agreed-upon standards on how to respond, and MOAs don’t always have the support from staff/physicians. • MOAs don’t always feel confident in how to approach specific patient communities (e.g., LGBTQ2s+) with care	Encouraged, subsidized, incentivized training for MOAs on facilitating caring relationships with patients - including how to deal with harassment, how to diffuse situations, compassion for patients with various experiences and communities (e.g. LGBTQ2s+, mental health). This training would be supported by: • Clear procedures in office on how to respond to challenging encounters, including guidance on when and how managers and physicians should step in • Centralized list of existing trainings on these topics for MOAs with clear guidance on how to access and funding available.	• Identify existing trainings on these topics and create a centralized list for clinics to access. Develop trainings where gaps exist • Incentives/funding for clinics to take trainings • Provincial support to develop in-clinic procedures on staff safety. • Training for managers and physicians on how to support MOAs when faced with harassment.

Provincial-wide MOA network: Supporting, advocating for, and advancing the interests of MOAs		
Problem	Solution	How
• MOAs feel isolated and disconnected from other MOAs • They have no where to turn to for guidance, resources, professional development, and training. • MOAs face challenges with navigating technological and administrative changes, and have little support to help them adapt • MOAs feel under recognized and under valued in their role	The association would support professional development, improve working conditions, advocate for recognition of MOAs, and ensure consistent standards across the province. Specifically: • Advocate for the recognition of MOAs as integral members of healthcare teams and push for improved wages, benefits, and working conditions. • Act as a resource for MOAs with updates on new laws and regulations • Foster a sense of community among MOAs by providing platforms for networking and sharing best practices. • Advocate for MOAs within healthcare policy discussions, ensuring that their voices are heard in decisions that affect their work environment and role within the healthcare system. Provide ongoing professional development opportunities, including workshops, webinars, and conferences on topics like medical terminology, EHR systems, and patient communication.	• Engage MOAs across the province and identify key issues • Partner with unions and associations • Figure out sustainable funding • Engage with colleges and universities offering MOA programs to build partnerships • Work with the Ontario Ministry of Health

### Delphi consensus process

A total of 49 individuals representing diverse perspectives across primary care were identified and invited via email to participate. 32 participants agreed to participate and completed the first round, which included MOAs (*n* = 12), family physicians (*n* = 9), primary care researchers (*n* = 4), health care executives and managers (*n* = 7). 21 participants completed both rounds of the survey and included MOAs (*n* = 10), family physicians (*n* = 4), primary care researchers (*n* = 3), health care executives and managers (*n* = 4).

**Fig. 2:**
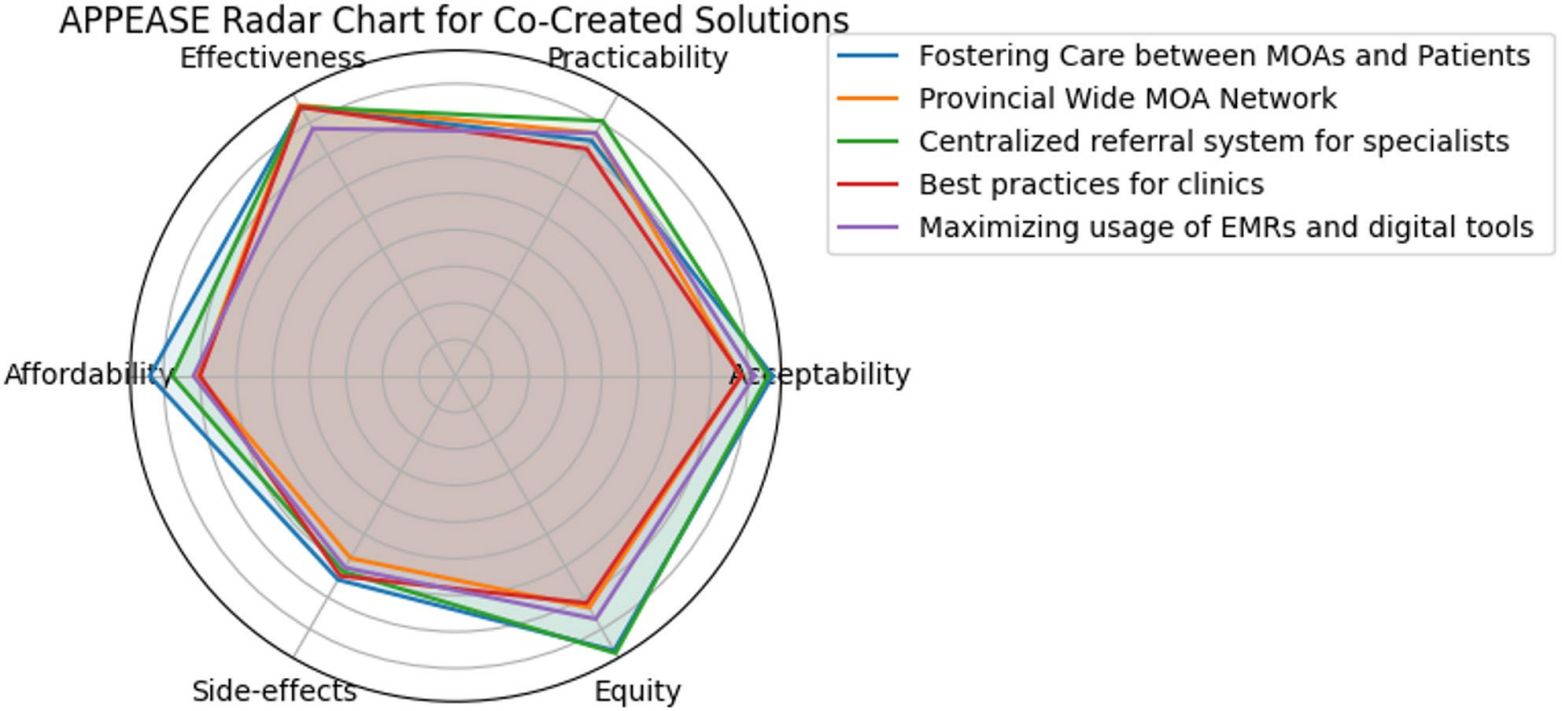
APPEASE Radar Chart for Co-Created Solutions

Results of the first survey where participants rated acceptability, practicability, effectiveness, affordability, potential for adverse effects, and equity for each solution can be found in Table 4. Full results are presented in Appendix 3, ranked by overall score, with disaggregated scores by participant type provided in Appendix 4. Overall, MOAs rated the solutions more favorably than other participant groups. Both MOAs and physicians identified the *Centralized Referral System* and *Fostering Care between MOAs and Patients* as their top two solutions. Health system leaders similarly ranked *Fostering Care between MOAs and Patients* as the most promising intervention. In the second survey respondents ranked the solutions again according to effectiveness, feasibility (i.e., acceptability, practicality, affordability), equity and side effects, and then gave an overall ranking (Table 4; Fig. 2, and Appendix 5 for full results).

**Table 4:** Delphi results from round 1 and round 2 of survey

Co-created solutions	APEASE* Surface Area *n* = 32	Part 1 Rank* n = 32	Part 2 Rank* n = 21
Fostering Care between MOAs and Patients×	41.60	1	1
Provincial Wide MOA Network×	35.32	5	2
Centralized referral system for specialists	41.19	2	3
Best practices for clinics×	34.91	6	4
Maximizing usage of EMRs and digital tools ×	35.93	3	5
Clinic-based education initiative	35.59	4	6
Patient Navigation Specialist	30.39	7	7
Province-wide Health-Care System Education Campaign	28.10	8	8

* APEASE scores are interpreted by plotting each criterion on a radar chart, where higher scores on each axis create a larger surface area, indicating an intervention that performs better overall across affordability, practicability, effectiveness, acceptability, safety, and equity

× Co-created solutions that were discussed in the final co-creation workshop

In open-text feedback, participants expanded on their APPEASE ratings for top-ranked solutions. For the *province-wide MOA network*, concerns included lack of compensation, unionization risks, cost, and regional role variability. The *Fostering Care between MOAs and Patients* initiative was valued but seen as dependent on leadership, funding, and clinic culture. Training must be trauma and equity-informed, with attention to operational impacts like clinic coverage.

On the *Best Practices for Clinics* resource, stakeholders appreciated the guidance for optimizing MOA roles but flagged risks of “competency creep” if responsibilities expand without sufficient training or support. Structural limitations, particularly in smaller clinics, along with unclear funding and delivery models were cited as major barriers. For *EMR and digital tool adoption*, participants cited barriers including cost, poor usability, and digital inequities. While full adoption could improve efficiency, many tools lack interoperability and overwhelm existing workflows.

The *centralized referral system* was broadly supported for reducing administrative burden and improving transparency, but challenges remain around specialist buy-in, system usability, and equity.

### Multi-stakeholder co-creation workshop

Eighteen participants contributed to the multi-stakeholder co-creation workshop, including health system leaders (5), family physicians (5), and researchers (5), and MOAs (3). Participants were divided into 4 groups, and each group was given the opportunity to develop more detailed action plans for one of the top 4 ranked solution: Fostering Care between MOAs and Patients, Provincial Wide MOA Network, Best practices for clinics, Maximizing usage of EMRs and digital tools. We chose not to include the high-scoring “Centralized Referral System” in the workshop as it represented a complex, system-level solution beyond the scope of primary care. Instead, the team opted to focus on MOA-centered solutions that aligned more closely with participants’ expertise and the practical realities of their primary care contexts, ensuring a more meaningful and grounded use of their time and insights. Final discussions emphasized the need for leadership engagement, clinic and system-level buy-in, and dedicated funding to support implementation. The final solutions from the workshops can be found in Table 2.

## Discussion

This study examined the experiences of MOAs in primary care and co-developed scalable solutions to support their evolving role. While MOAs described facing visible operational challenges, such as heavy workloads, complex patient interactions, and inefficient system processes, our analysis found that these are underpinned by deeper structural issues, including limited training, exclusion from decision-making, and undervaluation of their work. Together, these factors contribute to burnout and a sense of invisibility. Views of MOA work as “simple” or “clerical” further reinforce their marginalization. In response, MOAs identified and co-created eight solutions that targeted clinic-level and system-level improvements, including professional development initiatives, patient education campaigns, and structural changes such as centralized referral systems.

Our findings underscore the central yet often undervalued role that MOAs play in primary care clinics. Our research suggests that the heavy emotional and administrative burdens placed on MOAs, coupled with inadequate support structures, are not merely operational issues but symptoms of deeper societal views on the value of different kinds of work. While this interpretation is exploratory, it aligns with literature describing how such labour is often rendered invisible or undervalued within healthcare systems [22, 23]. These challenges are further compounded by entrenched power dynamics within primary care that limit MOAs’ autonomy, voice, and influence in decision-making processes [24]. MOAs collaboratively developed a series of proposed solutions to address the challenges identified in their roles. These solutions were subsequently assessed by a diverse group of stakeholders, including MOAs, family physicians, clinic managers, and system-level leaders, who evaluated them based on feasibility, acceptability, and potential equity impacts, and discussed strategies for implementation.

These results confirm and expand upon existing literature showing that MOAs are critical to the efficient operation of clinics yet remain largely unrecognized in health system planning [5, 25, 26]. This study directly engaged MOAs in co-creating pragmatic, scalable solutions, ensuring that interventions are grounded in the lived realities of MOAs. This participatory approach both empowers MOAs and increases the relevance and feasibility of the proposed solutions. Importantly, this reflects a broader recognition among professionals, MOAs, and other stakeholders of the need for solutions that are not only practical but that also leverage the full potential of MOAs to enhance the quality of care in primary care settings.

Three solutions were rated particularly highly. The first was the establishment of a professional association for MOAs, intended to support professional development, standardize training opportunities, and enhance recognition of MOAs within healthcare teams. The second prioritized the development of training programs and clinic procedures aimed at improving interactions between MOAs and patients, with an emphasis on addressing harassment, supporting mental health, and fostering equity-informed communication practices [24]. The third was the creation of a best practices guide for clinic operations, with the aim of improving workflow efficiency, clarifying roles, and optimizing the use of digital tools.

These findings can also be interpreted through the Job Demands–Resources (JD-R) framework, which suggests that employee wellbeing depends on the balance between job demands and the resources available to meet them [27, 28]. In our study, MOAs described heavy emotional and administrative demands alongside limited training, influence, and support, which are conditions that the JD-R model links to burnout and turnover [27, 28]. The co-created solutions may strengthen key job resources, including training, clearer processes, and greater recognition, which may enhance MOA wellbeing, satisfaction, and retention. Across several jurisdictions, primary care redesign initiatives are beginning to recognize the vital role of Medical Office Assistants. For example, in the United Kingdom and Australia, team-based models of care have expanded the roles of administrative staff to increasingly involve patient communication, managing digital records, care navigation, and supporting triage [29, 30]. Similarly, in parts of the United States and Canada, patient-centered medical homes (PCMHs) have integrated administrative staff as core members of the care team, often supported by standardized training and clear role definitions. In the Netherlands, practice assistants have been integrated into primary care teams to support general practitioners with both administrative and clinical responsibilities, including chronic disease management, lifestyle counselling, and care coordination [31]. This model has contributed to more efficient care delivery and enhanced access by redistributing routine tasks and allowing physicians to focus on complex patient needs [32, 33]. These international efforts mirror our focus on professionalizing the MOA role, enhancing training, and embedding MOAs more meaningfully in team-based care.

Several limitations must be acknowledged. First, the sample size, while diverse, was drawn from a single provincial context (Ontario), which may limit the generalizability of findings to other regions, although engagement with a broad range of stakeholders may increase their applicability within Ontario. However, Ontario is a province of over 16 million people and includes urban, rural, and remote primary care clinics. Further, the MOAs in our study were recruited from a wide scope of practice settings including small practice, large practice, community health center, and rural practice. Unfortunately, although many MOAs initially expressed interest in participating in a workshop, only nine ultimately confirmed, likely due to the shift from a general expression of interest in the survey to an invitation for a four-part series of 1.5-hour sessions, a significant time commitment. Second, participants were self-selected, which could introduce a bias toward MOAs who are more engaged or motivated to advocate for change. Third, while the co-created solutions were evaluated through a Delphi process with stakeholders, they have not yet been implemented or tested in practice, and their real-world effectiveness remains to be determined. Future research should focus on further co-designing, piloting, and evaluating the proposed solutions in diverse primary care settings to assess their feasibility, effectiveness, and scalability. Additionally, further exploration of MOAs’ experiences in rural, remote, and marginalized communities would be valuable, as these contexts may present unique challenges and opportunities. Taken together, this study should be viewed as an exploratory co-design initiative that generates promising, user-informed ideas for service improvement, which now require piloting and evaluation in broader and varied primary care settings.

## Conclusions

Medical Office Assistants are indispensable yet under-recognized members of primary care teams. Our findings highlight the multifaceted challenges MOAs face and point to systemic reforms needed to empower them to contribute fully and sustainably to primary care delivery. Investing in MOA recognition, training, support, and system-level reforms can be an opportunity to strengthen primary care, improve patient experiences, and build a more resilient health system.

## Supplementary Information

Supplementary Material 1.

Supplementary Material 2.

Supplementary Material 3.

## Data Availability

The datasets generated and analyzed during this study (including workshop notes, transcripts, and Delphi survey responses) contain information that could compromise the privacy of individual participants and therefore cannot be shared publicly. De-identified excerpts relevant to the findings are included in the article and its appendices. Additional data may be available from the corresponding author upon reasonable request and with approval from the Women’s College Hospital Research Ethics Board. Study protocol and consents are available upon request.

## Abbreviations

MOA: Medical office assistant
EMR: Electronic medical record
PCP: Primary care physician/provider
APEASE: Acceptability, practicability, effectiveness, affordability, safety, equity
OHIP: Ontario health insurance plan
RPN: Registered practical nurse
