# Supplementary material for: Elevating an invisible role: co-designing solutions to optimize medical office assistants in primary care
Source: BMC Prim Care. 2026 Jan 6;27:55. doi: 10.1186/s12875-025-03155-8 (PMC12892512; doi:10.1186/s12875-025-03155-8)
Supplement: Supplementary file 1 — Supplementary Material 1. [file 12875_2025_3155_MOESM1_ESM.docx]

**CONSENT**

**Study Title**: *Supporting the Face of Primary Care*

**Principal Investigator**: Jennifer Shuldiner, [*Jennifer.shuldiner@wchospital.ca*](mailto:Jennifer.shuldiner@wchospital.ca)

**Study Team/Research Contact**: Apira Ragunathan, [**apira.ragunathan@wchospital.ca**](mailto:apira.ragunathan@wchospital.ca)

**Study Sponsor/Funder(s):** *Women’s College Hospital, Canadian Institute for Health Research*

INTRODUCTION

Thank you for considering taking part in the second round of this 2-part Delphi survey (a series of surveys in which expert opinions are gathered on a topic to gain consensus). The purpose of this survey is to explore and establish a consensus on potential solutions to address key challenges faced by medical office assistants in primary care settings. The results of this survey will be used to guide our discussion during the policy workshop, in which we will co-create solutions based on the feasibility and prioritization determined from this survey.

You have the option to consent and continue with the survey below.

IS THERE A CONFLICT OF INTEREST? There are no conflicts of interest to declare related to this study.

WHAT WILL HAPPEN DURING THIS STUDY AND WHAT ARE THE RESPONSIBILITIES OF STUDY PARTICIPANTS? This survey will take about 15-20 minutes to complete. In this survey, you will be presented with the results of round 1 of this survey, and asked rank all the solutions.

CAN PARTICIPANTS CHOOSE TO LEAVE THE STUDY? You can choose to end your participation in the study (called withdrawal) at any time without having to provide a reason.

WHAT ARE THE RISKS OR HARMS OF PARTICIPATING IN THIS STUDY? The risks associated with your participation is this study are very low. The study team will follow all of the measures described in this consent form to keep your information confidential. The information you provide in the survey will not be shared with anyone at your workplace. However, even though the likelihood that someone may identify you from the study data is very small, it can never be completely eliminated.

WHAT ARE THE BENEFITS OF PARTICIPATING IN THIS STUDY? There are no direct personal benefits to participating in this project. By participating, your opinions and ideas may be incorporated into future implementation and research initiatives regarding primary care and medical office assistants.

HOW WILL PARTICIPANT INFORMATION BE KEPT CONFIDENTIAL? All data will be stored securely on encrypted, password-protected servers in Women’s College Hospital in Toronto, Ontario, Canada. These offices are locked and, in a swipe-card protected access area. Electronic copies of the survey responses will be archived in a secure, password protected hospital server for a period of 10 years. Data will only be accessed by REB-approved team members.
 
Your answers will only be used for study purposes by Women's College Hospital research team. All information collected will be kept confidential. Responses will only be presented in aggregate. You will be asked for your email address so that we may send you your honorarium and part two of the survey. The project staff, the WCH Research Ethics Board, and employees of the sponsor or funder of the project may look at your personal information for purposes associated with the project. The members of the study team and Women’s College Hospital (WCH) will be obligated to protect your privacy and not disclose your personal information. In addition, your name will not be denoted in any project records, except the survey participation log which will be stored separately from all other project records. Any information reported in presentations, formal reports, or publications will not disclose your personal identity or identifying information (organization etc.).

ARE STUDY PARTICIPANTS PAID TO BE IN THIS STUDY? You will receive an honorarium of $50 e-gift card as a thank you after completion of *both rounds* of the Delphi survey.

WHAT ARE THE RIGHTS OF PARTICIPANTS IN A RESEARCH STUDY? You have the right to be informed of the results of this study once the entire study is complete. We estimate the results of this study will be available in 1 year. The team will provide a report summarizing the results of the study. Your rights to privacy are legally protected by federal and provincial laws that require safeguards to ensure that your privacy is respected.

By signing this form you do not give up any of your legal rights against the researcher/study doctor, sponsor or involved institutions for compensation, nor does this form relieve the researcher/study doctor, sponsor or their agents of their legal and professional responsibilities.

WHOM DO PARTICIPANTS CONTACT FOR QUESTIONS? If you have questions about taking part in this study, or if you suffer a research-related injury, you can talk to the research team, or the person who is in charge of the study at this institution. That person is: Jennifer Shuldiner: [jennifer.shuldiner@wchospital.ca](mailto:jennifer.shuldiner@wchospital.ca)

Research Ethics Board Contact: If you have questions about your rights as a participant or concerns about ethical issues related to this study, you can talk to someone who is not involved in the study at all. That person is the Chair of the Research Ethics Board, who can be contacted at [ethics@wchospital.ca](mailto:ethics@wchospital.ca), or you can leave a message at 416-351-3732 ext. 2325.

**Do you consent to participation in this survey?**
☐ Yes ☐ No

**Name:** ________________________________

**Today’s Date:** __________________________

**What province do you work in?** ____________________

**Which group best represents your perspective? (select all that apply)**

Physician (primary care provider)

Nurse

MOA

Other (please specify): __________________

**What is your email so we can provide your honorarium for completing this 2-part survey?**

__________________

**Thinking of the effectiveness of each solution, rank the following solutions from 1-8, with 1 being highest *effectiveness* and 8 being the *lowest*.**

**Drag the solutions to rank them:**

1- Provincial-wide MOA network: Supporting, advocating for, and advancing the interests of MOAs [average = 4.3]

2- Care between MOAs and Patients: Training and procedures to support safety and enabling caring relationships [average = 4.3]

3- Centralized referral system for specialists: Seamless specialist referral and simplified care coordination [average = 4.3]

4- Best practices for clinics: Helping physicians manage their clinic and make the most of MOAs [average = 4.3]

5- Maximizing usage of EMRs and digital tools: Training, support and incentives for full adoption [average = 3.9]

6- Clinic-based education initiative: Educating patients on clinic process, roles, and expectations to lower MOA burden and build shared understanding [average = 3.9]

7- Patient Navigation Specialist: A new role to simplify health care access [average = 3.5]

8- Province-wide Health-Care System Education Campaign: Empowering patients to navigate the system and lower the burden on MOAs [average = 3.2]

**Why did you rank this way?** _________________________

**Thinking of the feasibility of each solution, rank the following solutions from 1-8, with 1 being most *feasible*.**

**Drag the solutions to rank them:**

1- Fostering Care between MOAs and Patients: Training and procedures to support safety and enabling caring relationships [average = 4.1]

2- Centralized referral system for specialists: Seamless specialist referral and simplified care coordination [average = 4.1]

3 - Clinic-based education initiative: Educating patients on clinic process, roles, and expectations to lower MOA burden and build shared understanding [average = 3.8]

4 - Maximizing usage of EMRs and digital tools: Training, support and incentives for full adoption [average = 3.8]

5- Provincial-wide MOA network: Supporting, advocating for, and advancing the interests of MOAs [average= 3.7]

6- Best practices for clinics: Helping physicians manage their clinic and make the most of MOAs [average= 3.7]

7- Patient Navigation Specialist: A new role to simplify health care access [average = 3.5]

8- Province-wide Health-Care System Education Campaign: Empowering patients to navigate the system and lower the burden on MOAs [average = 3.4]

**Why did you rank this way?** _________________________

**Thinking of the side-effects of each solution, rank the following solutions from 1-8, with 1 being the least amount of *unintended side-effects.***

**Drag the solutions to rank them.**

1- Fostering Care between MOAs and Patients: Training and procedures to support safety and enabling caring relationships [average = 3.2]

2- Best practices for clinics: Helping physicians manage their clinic and make the most of MOAs [average = 3.2]

3- Centralized referral system for specialists: Seamless specialist referral and simplified care coordination [average = 3.1]

4- Province-wide Health-Care System Education Campaign: Empowering patients to navigate the system and lower the burden on MOAs [average = 3.0]

5- Maximizing usage of EMRs and digital tools: Training, support and incentives for full adoption [average = 3.0]

6- Clinic-based education initiative: Educating patients on clinic process, roles, and expectations to lower MOA burden and build shared understanding [average = 2.9]

7- Provincial-wide MOA network: Supporting, advocating for, and advancing the interests of MOAs [average = 2.9]

8- Patient Navigation Specialist: A new role to simplify health care access [average =2.8]

**Why did you rank this way?** _________________________

**Thinking about equity considerations for each solution, rank the following solutions from 1-8, with 1 being the most *equitable*.**

**Drag the solutions to rank them.**

1 - Centralized referral system for specialists: Seamless specialist referral and simplified care coordination [average = 4.4]

2 - Fostering Care between MOAs and Patients: Training and procedures to support safety and enabling caring relationships [average = 4.3]

3 - Patient Navigation Specialist: A new role to simplify health care access [average = 3.9]

4 - Maximizing usage of EMRs and digital tools: Training, support and incentives for full adoption [average =3.8]

5 - Clinic-based education initiative: Educating patients on clinic process, roles, and expectations to lower MOA burden and build shared understanding [average = 3.8]

6 - Provincial-wide MOA network: Supporting, advocating for, and advancing the interests of MOAs [average = 3.7]

7 - Best practices for clinics: Helping physicians manage their clinic and make the most of MOAs [average = 3.6]

8 - Province-wide Health-Care System Education Campaign: Empowering patients to navigate the system and lower the burden on MOAs [average = 3.3]

Why did you rank this way? _________________________

**Thinking about all domains, please rank the following solutions from 1-8, with 1 being the *highest priority*.**

**Drag the solutions to rank them.**

1- Fostering Care between MOAs and Patients: Training and procedures to support safety and enabling caring relationships [average = 4.0]

2 - Centralized referral system for specialists: Seamless specialist referral and simplified care coordination [average = 4.0]

3 - Provincial-wide MOA network: Supporting, advocating for, and advancing the interests of MOAs [average = 3.7]

4 - Best practices for clinics: Helping physicians manage their clinic and make the most of MOAs [average = 3.7]

5 - Clinic-based education initiative: Educating patients on clinic process, roles, and expectations to lower MOA burden and build shared understanding [average = 3.6]

6 - Maximizing usage of EMRs and digital tools: Training, support and incentives for full adoption [average = 3.6]

7 - Patient Navigation Specialist: A new role to simplify health care access [average = 3.5]

8 - Province-wide Health-Care System Education Campaign: Empowering patients to navigate the system and lower the burden on MOAs [average = 3.2]

**Why did you rank this way?** _________________________

Thank you for completing this survey!
