# Supplementary material for: Elevating an invisible role: co-designing solutions to optimize medical office assistants in primary care
Source: BMC Prim Care. 2026 Jan 6;27:55. doi: 10.1186/s12875-025-03155-8 (PMC12892512; doi:10.1186/s12875-025-03155-8)
Supplement: Supplementary file 2 — Supplementary Material 2. [file 12875_2025_3155_MOESM2_ESM.docx]

**CONSENT**

**Study Title**: *Supporting the Face of Primary Care*

**Principal Investigator**: Jennifer Shuldiner, [*Jennifer.shuldiner@wchospital.ca*](mailto:Jennifer.shuldiner@wchospital.ca)

**Study Team/Research Contact**: Apira Ragunathan, [**apira.ragunathan@wchospital.ca**](mailto:apira.ragunathan@wchospital.ca)

**Study Sponsor/Funder(s):** *Women’s College Hospital, Canadian Institute for Health Research*

INTRODUCTION

Thank you for considering taking part in this two-round Delphi survey (a series of surveys in which expert opinions are gathered on a topic to gain consensus). The purpose of this survey is to explore and establish a consensus on potential solutions to address key challenges faced by medical office assistants in primary care settings.

The results of this survey will be used to guide our discussion during the policy workshop, in which we will co-create solutions based on the feasibility and prioritization determined from this survey.

You are also invited to attend this workshop, and attendance is voluntary. If you are interested, please contact the study coordinator at apira.ragunathan@wchospital.ca.

IS THERE A CONFLICT OF INTEREST? There are no conflicts of interest to declare related to this study.

WHAT WILL HAPPEN DURING THIS STUDY AND WHAT ARE THE RESPONSIBILITIES OF STUDY PARTICIPANTS? This survey will take about 15-20 minutes to complete. You may complete the first part of the survey on the following pages, and the link to complete the second part of the survey will be emailed to you on November 20^th^. In this survey, you will be presented with 8 initiatives, detailing the problem, solution and how it can be achieved. You will be asked your opinion on statements relating to these initiatives based on the APPEASE criteria (Acceptability, Practicability, Effectiveness, Affordability, Side-effects, and Equity), by rating them on a Likert scale (strongly agree, agree, neutral, disagree, strongly disagree).

CAN PARTICIPANTS CHOOSE TO LEAVE THE STUDY? You can choose to end your participation in the study (called withdrawal) at any time without having to provide a reason.

WHAT ARE THE RISKS OR HARMS OF PARTICIPATING IN THIS STUDY? The risks associated with your participation is this study are very low. The study team will follow all of the measures described in this consent form to keep your information confidential. The information you provide in the survey will not be shared with anyone at your workplace. However, even though the likelihood that someone may identify you from the study data is very small, it can never be completely eliminated.

WHAT ARE THE BENEFITS OF PARTICIPATING IN THIS STUDY? There are no direct personal benefits to participating in this project. By participating, your opinions and ideas may be incorporated into future implementation and research initiatives regarding primary care and medical office assistants.

HOW WILL PARTICIPANT INFORMATION BE KEPT CONFIDENTIAL? All data will be stored securely on encrypted, password-protected servers in Women’s College Hospital in Toronto, Ontario, Canada. These offices are locked and, in a swipe-card protected access area. Electronic copies of the survey responses will be archived in a secure, password protected hospital server for a period of 10 years. Data will only be accessed by REB-approved team members.
 
Your answers will only be used for study purposes by Women's College Hospital research team. All information collected will be kept confidential. Responses will only be presented in aggregate. You will be asked for your email address so that we may send you your honorarium and part two of the survey. The project staff, the WCH Research Ethics Board, and employees of the sponsor or funder of the project may look at your personal information for purposes associated with the project. The members of the study team and Women’s College Hospital (WCH) will be obligated to protect your privacy and not disclose your personal information. In addition, your name will not be denoted in any project records, except the survey participation log which will be stored separately from all other project records. Any information reported in presentations, formal reports, or publications will not disclose your personal identity or identifying information (organization etc.).

ARE STUDY PARTICIPANTS PAID TO BE IN THIS STUDY? You will receive an honorarium of $50 e-gift card as a thank you after completion of *both rounds* of the Delphi survey.

WHAT ARE THE RIGHTS OF PARTICIPANTS IN A RESEARCH STUDY? You have the right to be informed of the results of this study once the entire study is complete. We estimate the results of this study will be available in 1 year. The team will provide a report summarizing the results of the study. Your rights to privacy are legally protected by federal and provincial laws that require safeguards to ensure that your privacy is respected.

By signing this form you do not give up any of your legal rights against the researcher/study doctor, sponsor or involved institutions for compensation, nor does this form relieve the researcher/study doctor, sponsor or their agents of their legal and professional responsibilities.

WHOM DO PARTICIPANTS CONTACT FOR QUESTIONS? If you have questions about taking part in this study, or if you suffer a research-related injury, you can talk to the research team, or the person who is in charge of the study at this institution. That person is: Jennifer Shuldiner: [jennifer.shuldiner@wchospital.ca](mailto:jennifer.shuldiner@wchospital.ca)

Research Ethics Board Contact: If you have questions about your rights as a participant or concerns about ethical issues related to this study, you can talk to someone who is not involved in the study at all. That person is the Chair of the Research Ethics Board, who can be contacted at [ethics@wchospital.ca](mailto:ethics@wchospital.ca), or you can leave a message at 416-351-3732 ext. 2325.

Do you consent to participation in this survey?

Name:

Today’s date:

Yes, proceed

No

**BACKGROUND**

1. What is your email address?
2. What province?
3. What group best represents your perspective? (click on all that apply)
   1. Primary care provider
      1. Physician
      2. Nurse practitioner
   2. Primary care nurse
   3. policy maker
   4. government
   5. provincial association or health system executives
   6. MOA
   7. Patient
   8. Primary care researcher
4. How many years have you worked in this role/field?
5. What is your email so we can contact you regarding the second round of the survey and provide honorarium:

In this section, you will be presented with 8 concept cards that present policy solutions that address key challenges faced by medical office assistants in primary care.

For each proposed solution, please rate it using the 5-point scale which varies from strongly disagree to strongly agree, with a neutral option in the middle.

In addition to the ratings, you may provide comments regarding the challenges or possible solutions. Your input will help determine which solutions should be pursued.

**Concept Card 1: Maximizing the EMR and Digital Tools**


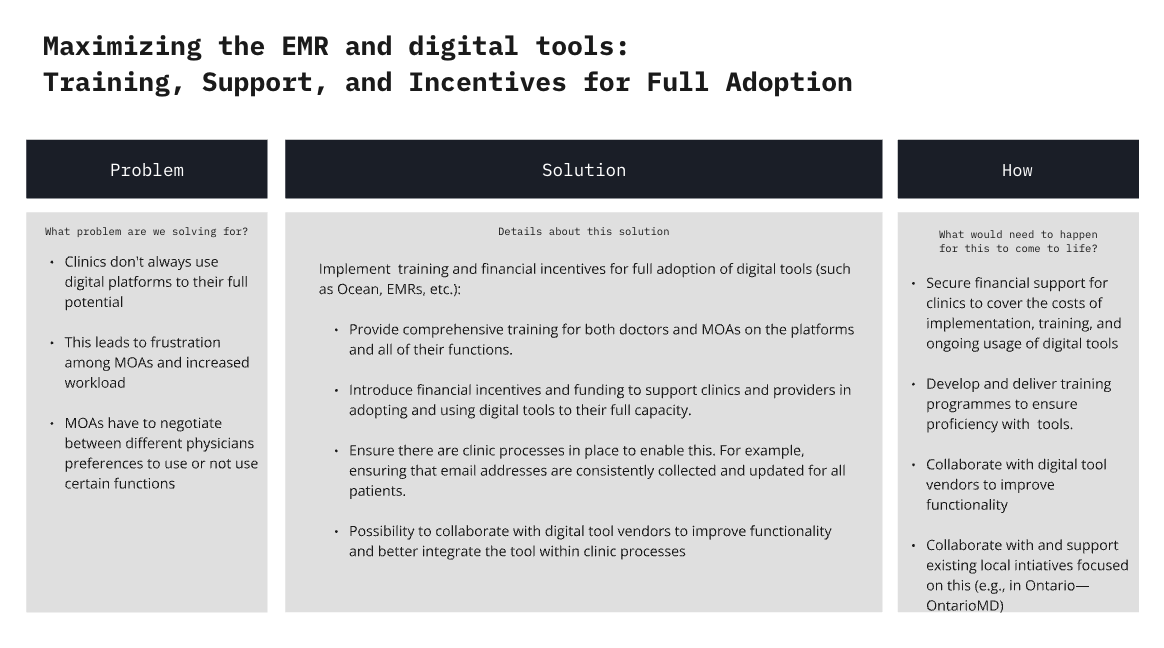


| **To what extent do you agree or disagree with the following statements?** | **Strongly Agree** | **Agree** | **Neutral** | **Disagree** | **Strongly Disagree** |
| --- | --- | --- | --- | --- | --- |
| The costs of training and incentives for full digital tool adoption are justified by the expected improvements in clinic efficiency. (affordability) |  |  |  |  |  |
| Clinics can incorporate comprehensive training for both MOAs and physicians in digital tools given current clinic structures and processes. (practicability) |  |  |  |  |  |
| This solution would effectively improve the use of digital tools and reduce MOA workload. (effectiveness) |  |  |  |  |  |
| Physicians and MOAs will be willing to fully adopt and integrate digital tools into their daily workflow. (acceptability) |  |  |  |  |  |
| This solution has unintended risks involved. (spillover effects) |  |  |  |  |  |
| This solution would promote equal access to digital tools across different clinics and regions. (Equity) |  |  |  |  |  |

1. Do you have any additional comments, suggestions, or concerns related to this issue that were not addressed in the previous questions?
2. What are the unintended consequences from implementing this program/policy?

**Concept Card 2: Centralized referral system for specialists**


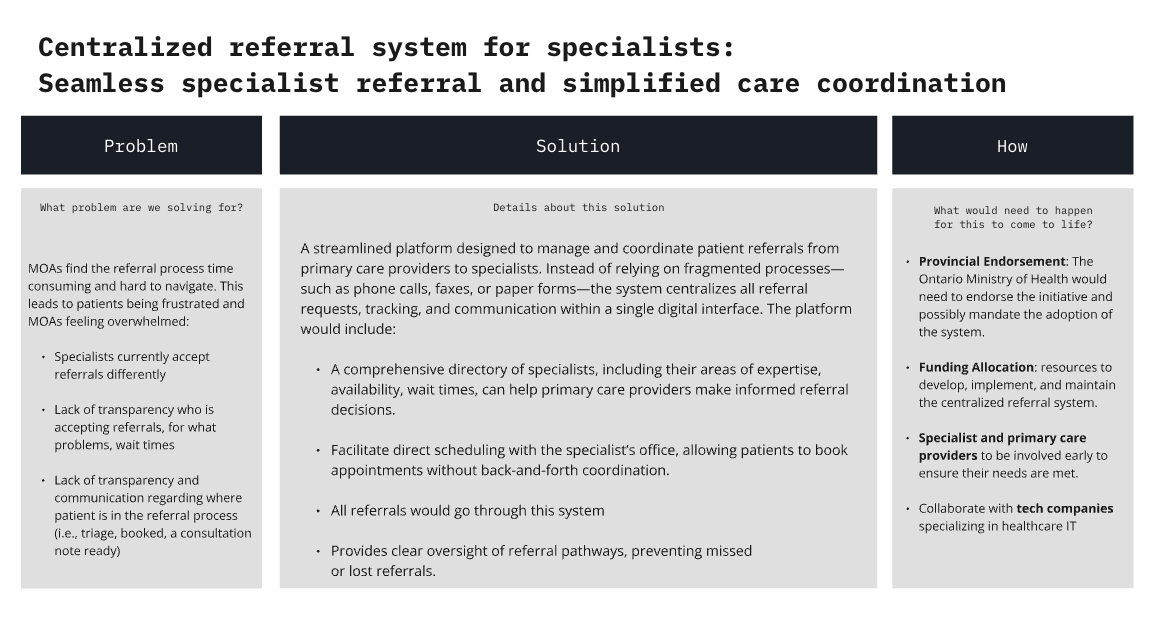


| **To what extent do you agree or disagree with the following statements?** | **Strongly Agree** | **Agree** | **Neutral** | **Disagree** | **Strongly Disagree** |
| --- | --- | --- | --- | --- | --- |
| The cost of implementing a centralized referral system is justified by the increased efficiency in managing specialist referrals. (affordability) |  |  |  |  |  |
| It will be feasible for clinics and specialists to adopt and consistently use a centralized referral system. |  |  |  |  |  |
| A centralized referral system will significantly reduce the time and workload involved in managing referrals for MOAs |  |  |  |  |  |
| Specialists, primary care providers, and patients will support the adoption of a centralized referral system. |  |  |  |  |  |
| This solution has unintended risks involved. |  |  |  |  |  |
| This solution would promote equal access to specialist care across different regions and clinic types. |  |  |  |  |  |

1. Do you have any additional comments, suggestions, or concerns related to this issue that were not addressed in the previous questions?
2. What are the unintended consequences from implementing this program/policy?

**Concept Card 3: Patient Navigation Specialist**


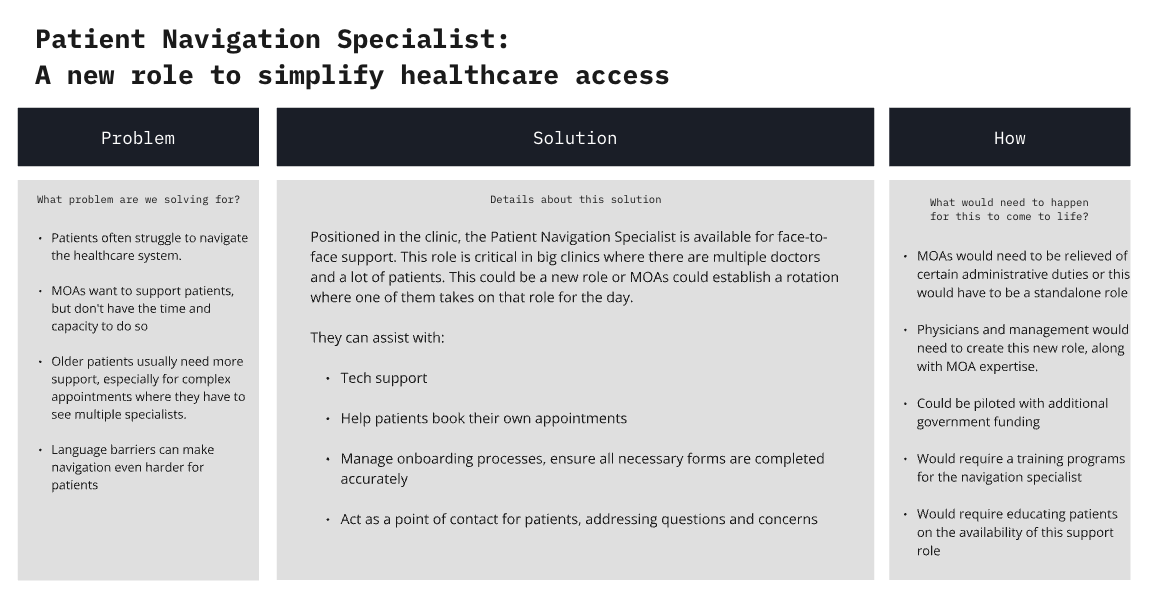


| **To what extent do you agree or disagree with the following statements?** | **Strongly Agree** | **Agree** | **Neutral** | **Disagree** | **Strongly Disagree** |
| --- | --- | --- | --- | --- | --- |
| The cost of creating this role or increasing MOA’s scope is reasonable given potential benefits. |  |  |  |  |  |
| It is practical for clinics to implement a patient navigation specialist role, given current staff capacity and workloads. |  |  |  |  |  |
| The introduction of a patient navigation specialist will significantly reduce MOA workload and improve patient satisfaction. |  |  |  |  |  |
| This solution would be acceptable to MOAs, physicians, and patients. |  |  |  |  |  |
| This solution has unintended risks involved. |  |  |  |  |  |
| This solution would promote equal access to navigation support for all patients. |  |  |  |  |  |

1. Do you have any additional comments, suggestions, or concerns related to this issue that were not addressed in the previous questions?
2. What are the unintended consequences from implementing this program/policy?

**Concept Card 4: Health-care Systems Navigation System**


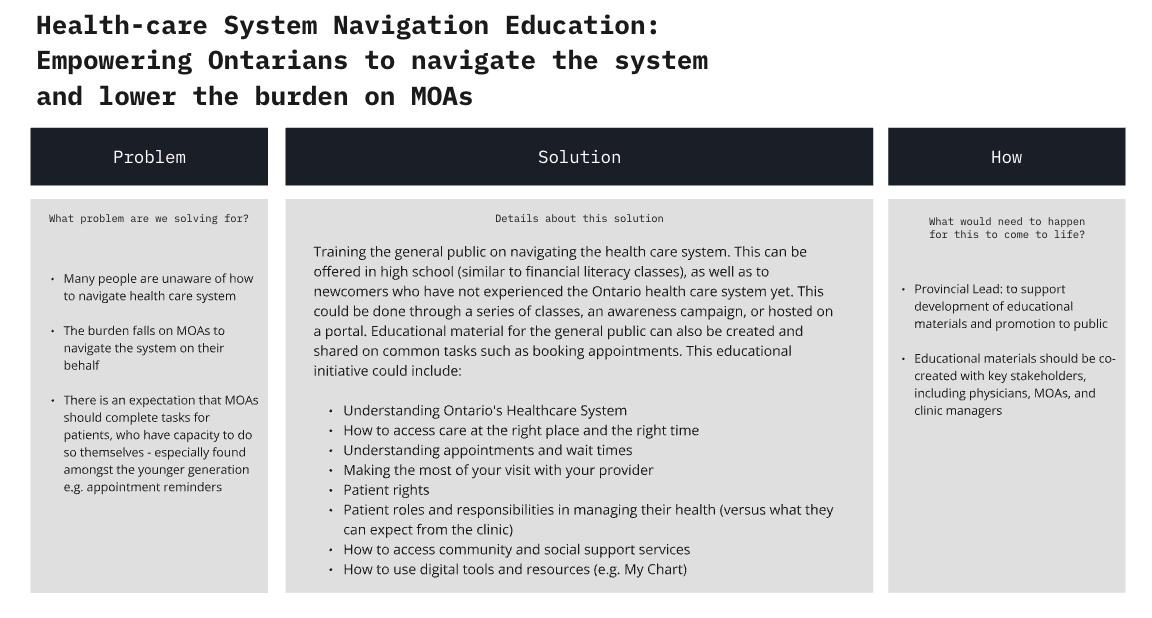


| **To what extent do you agree or disagree with the following statements?** | **Strongly Agree** | **Agree** | **Neutral** | **Disagree** | **Strongly Disagree** |
| --- | --- | --- | --- | --- | --- |
| The costs associated with developing and implementing a healthcare navigation education program for the public are justified by the long-term reduction in MOA workload. |  |  |  |  |  |
| The general public will be able to access and engage with the healthcare navigation materials. |  |  |  |  |  |
| Educating the public on how to navigate the healthcare system will significantly reduce the burden on MOAs and clinic staff. |  |  |  |  |  |
| The public will be receptive to learning about healthcare navigation and will apply the knowledge in their interactions with the system. |  |  |  |  |  |
| This solution has unintended risks involved. |  |  |  |  |  |
| This solution would promote equal understanding and access to healthcare for all individuals and therefore equally support MOAs from all regions and clinic types. |  |  |  |  |  |

1. Do you have any additional comments, suggestions, or concerns related to this issue that were not addressed in the previous questions?
2. What are the unintended consequences from implementing this program/policy?

**Concept Card 5: Clinic Clarity**


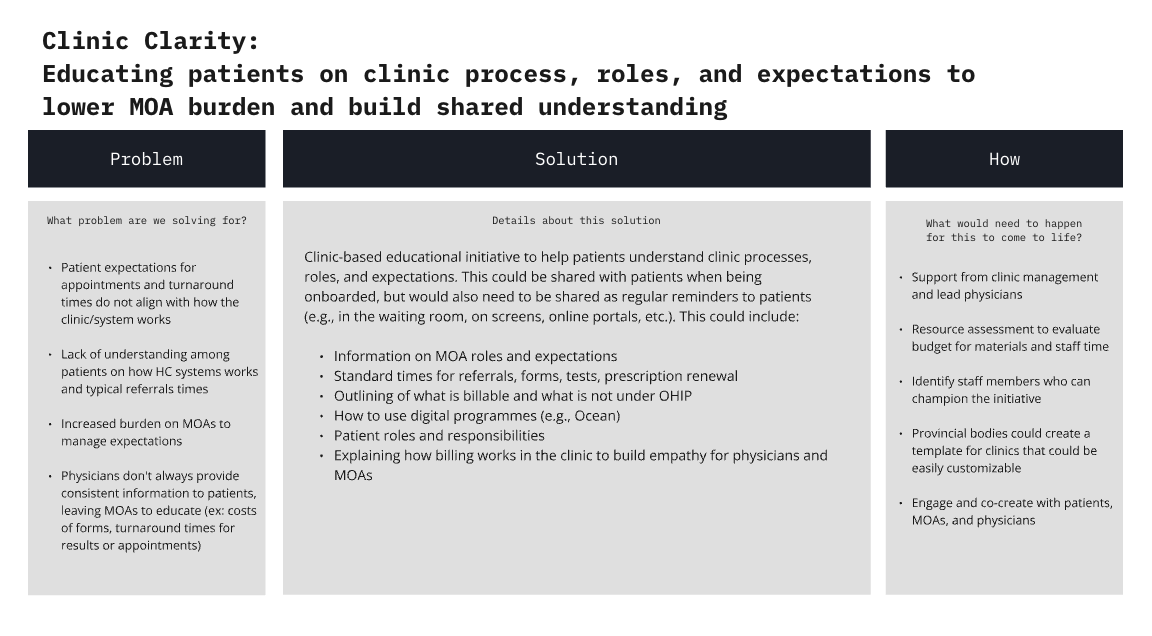


| **To what extent do you agree or disagree with the following statements?** | **Strongly Agree** | **Agree** | **Neutral** | **Disagree** | **Strongly Disagree** |
| --- | --- | --- | --- | --- | --- |
| The cost of developing and implementing these educational initiatives is justified by the expected reduction in MOA workload and patient confusion. |  |  |  |  |  |
| Educational materials can be easily integrated into existing clinic workflows, such as during onboarding or through digital platforms. |  |  |  |  |  |
| The solution will increase patients’ understanding of clinic procedures which will be beneficial to MOAs. |  |  |  |  |  |
| This solution would be acceptable to MOAs, physicians, and patients. |  |  |  |  |  |
| This solution has unintended risks involved. |  |  |  |  |  |
| This initiative will equally support MOAs from all regions and clinic types. |  |  |  |  |  |

1. Do you have any additional comments, suggestions, or concerns related to this issue that were not addressed in the previous questions?
2. What are the unintended consequences from implementing this program/policy?

**Concept 6: MOAs are essential to primary care teams**
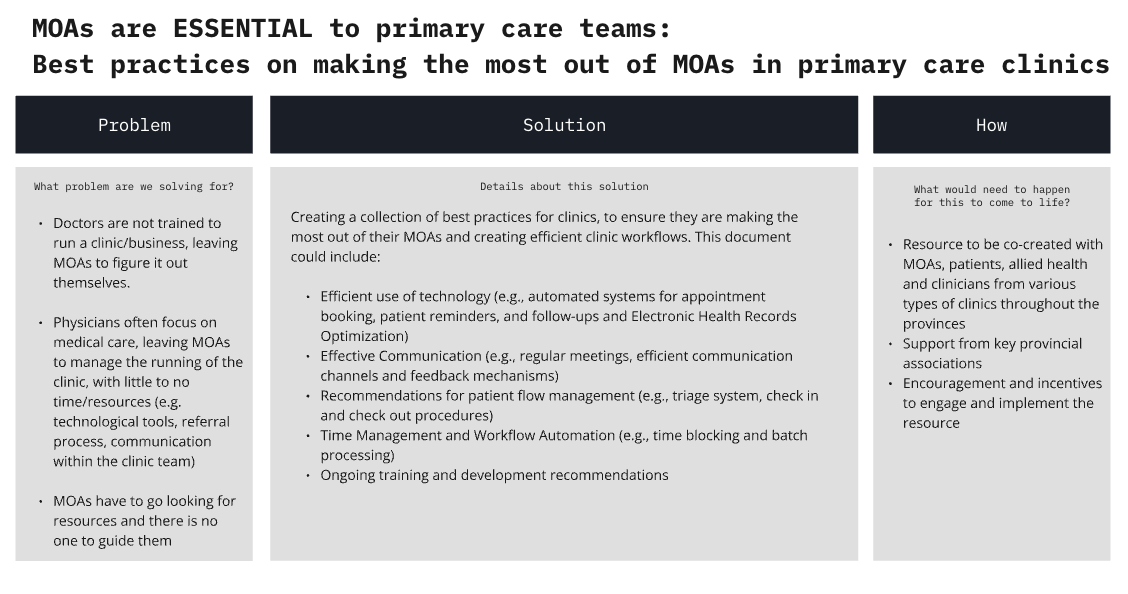


| To what extent do you agree or disagree with the following statements? | **Strongly Agree** | **Agree** | **Neutral** | **Disagree** | **Strongly Disagree** |
| --- | --- | --- | --- | --- | --- |
| The development of a best practices guide for making the most of MOAs in clinics is a cost-effective way to improve clinic efficiency. |  |  |  |  |  |
| This solution is feasible to implement across various clinic types and structures. |  |  |  |  |  |
| Optimizing the role of MOAs will increase efficiency of the clinic and be beneficial to MOAs. |  |  |  |  |  |
| Clinic management and physicians will be willing to adopt and support the implementation of best practices for MOAs. |  |  |  |  |  |
| This solution has unintended risks involved. |  |  |  |  |  |
| The best practices guide will benefit all clinics, regardless of size or location, in optimizing their use of MOAs. |  |  |  |  |  |

1. Do you have any additional comments, suggestions, or concerns related to this issue that were not addressed in the previous questions?
2. What are the unintended consequences from implementing this program/policy?

**Concept Card 7: Fostering care between MOAs and patients**


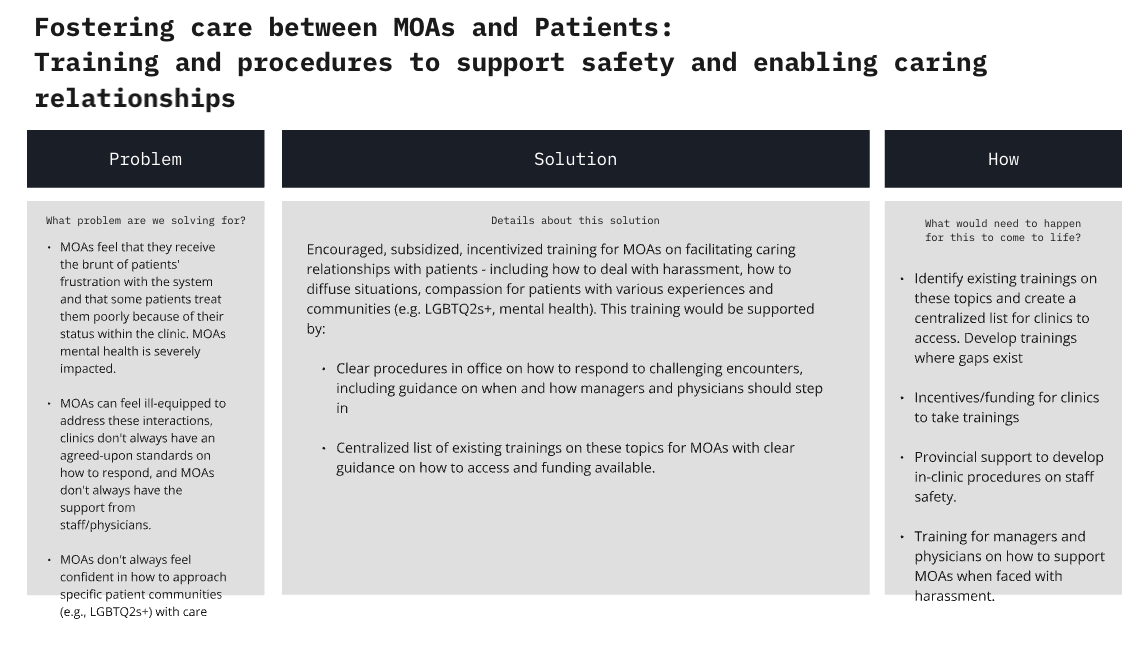


| **To what extent do you agree or disagree with the following statements?** | **Strongly Agree** | **Agree** | **Neutral** | **Disagree** | **Strongly Disagree** |
| --- | --- | --- | --- | --- | --- |
| The costs of providing training to MOAs on fostering caring relationships with patients are justified by the potential improvements in patient experience and MOA well-being. |  |  |  |  |  |
| It is practical for clinics to implement training programs for MOAs on handling challenging patient interactions and fostering compassionate care. |  |  |  |  |  |
| The solution will significantly improve MOAs ability to manage difficult situations and reduce stress. |  |  |  |  |  |
| This solution would be acceptable to MOAs and physicians. |  |  |  |  |  |
| This solution has unintended risks involved. |  |  |  |  |  |
| All clinics, regardless of size or location, will benefit equally from providing MOA training in patient care and communication. |  |  |  |  |  |

1. Do you have any additional comments, suggestions, or concerns related to this issue that were not addressed in the previous questions?
2. What are the unintended consequences from implementing this program/policy?

**Concept Card 8: Provincial-wide MOA network**


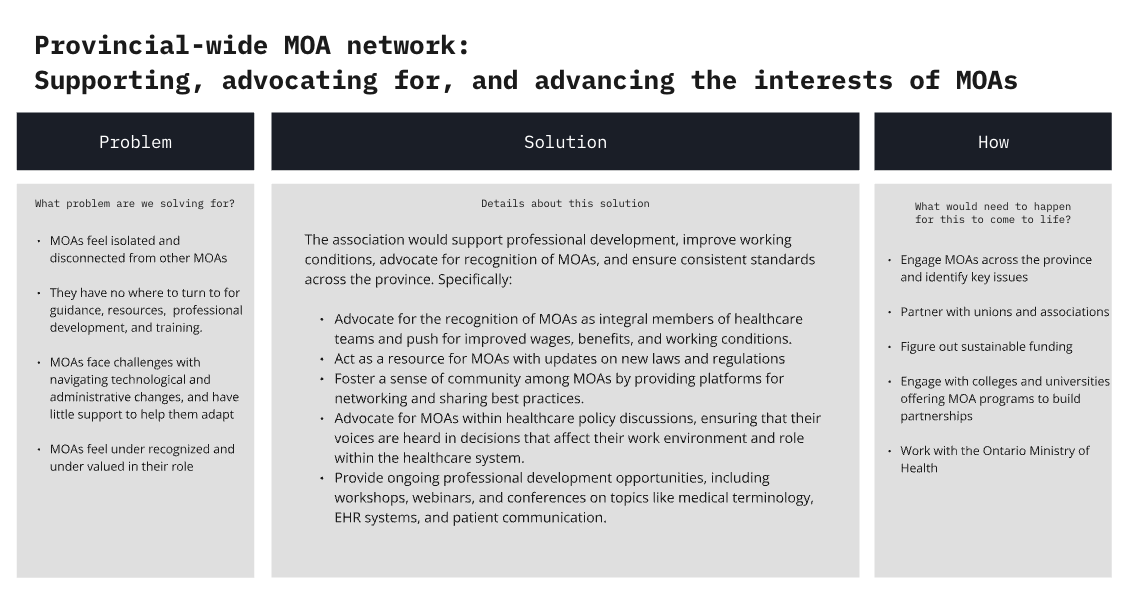


| **To what extent do you agree or disagree with the following statements?** | **Strongly Agree** | **Agree** | **Neutral** | **Disagree** | **Strongly Disagree** |
| --- | --- | --- | --- | --- | --- |
| Establishing a provincial-wide MOA network is a cost-effective way to support MOAs and improve working conditions. |  |  |  |  |  |
| It is practical to establish a provincial-wide MOA network to provide support, training, and advocacy for MOAs. |  |  |  |  |  |
| A provincial-wide MOA network will support MOAs and improve professional development and recognition. |  |  |  |  |  |
| MOAs will be willing to participate in a provincial network for professional development and peer support. |  |  |  |  |  |
| This solution has unintended risks involved. |  |  |  |  |  |
| A provincial-wide MOA network will equally support MOAs from all regions and clinic types. |  |  |  |  |  |

1. Do you have any additional comments, suggestions, or concerns related to this issue that were not addressed in the previous questions?
2. What are the unintended consequences from implementing this program/policy?

END OF SURVEY

**We thank you for the time you spent taking this survey. Your response has been recorded.**

**You will receive the second round of the Delphi survey on November 20th.**

**If you have any questions, please reach out to: apira.ragunathan@wchospital.ca**
