# Supplementary material for: Elevating an invisible role: co-designing solutions to optimize medical office assistants in primary care
Source: BMC Prim Care. 2026 Jan 6;27:55. doi: 10.1186/s12875-025-03155-8 (PMC12892512; doi:10.1186/s12875-025-03155-8)
Supplement: Supplementary file 3 — Supplementary Material 3. [file 12875_2025_3155_MOESM3_ESM.docx]

**Appendix 1: Discussion guides for MOA workshops and Final co-creation workshops**

**MOA workshops**

**Meeting 1**

| **Opening (10 min)** | Goals of the project  Outline of sessions  Invitations  How we will use your data/commitments |
| --- | --- |
| **Warm-up (20 min)** | Tell us your name  Group 1: Share a memorable story from your experience as an MOA  Group 2: Share a story about a patient interaction that felt good? |
| **Current Experience Mapping (25 min)** | "We want to start by better understanding your current experience as an MOA, specifically what is challenging/hard and what is pleasurable or meaningful. The idea is that by doing so, we can start to identify opportunities to make your experience at work better.   1. Facilitator to open space for open dialogue 2. Note taker takes notes under either challenging or meaningful 3. If/when specific ideas or solutions comes up, they can be put in the idea/opportunity garage   Additional prompts if people need help thinking broadly or deeply. Consider:   - your role - what you spend most time doing (or what you don't spend a lot of time on) - your relationships with other people (HCPs, other MOAs, patients, etc.) - the systems or tools you need to use - the types of supports available to you - your place of work - [if hasn't come up yet] - your role in primary care - What tasks or encounters with patients with might it help to have more training in? - Do you get a chance to make suggestions about how to make the clinic work better for you, for other staff, or for patients? |
| **Next Steps and Close**  **(5 min)** | 1. Thank you 2. Next meeting - we start thinking about what a more ideal MOA experience could look like 3. The Miro board is always open - you can add more or edit as you like. 4. We would also love to hear your thoughts and feedback - what worked, what didn't and why - you can add it on the Miro or email us at anytime |

**Meeting 2**

| **Opening (5 min)** | "Last time we spent time understanding the current state - what is working, what isn't: we heard….    Today, we hope to think about the future and explore what would a more ideal, or more meaningful work experience look like." |
| --- | --- |
| **Warm-up (10 min)** | We are going to spend some time envisioning what an ideal day as an MOA looks like. Let's practice envisioning ideal together.    Facilitator to begin with their vision and ensure that there a lot of detail and storytelling    Give people 2 minutes silent time to think    While thinking ask the following questions: What would your ideal summer day look like?   - How would it begin? - How would you feel? - What would you do? Not do? - Who would be there? - How would it end? |
| **Envisioning an Ideal Day (45 min)** | "Let's slow down and imagine a day in the life where we have made changes so that your work feels more meaningful. Think about what an ideal day at work would feel/look like - how does it start, what happens next, what doesn't happen, etc."   1. Explain why this is helpful: "We are asking you to think about what would be ideal, because often we get stuck in feasible. We think about what is possible vs what is desirable. And the hope is that we can think of solutions that get us to desirable not just possible. In other words, we can't bring innovative things to life, if we can't even imagine it is possible. Let's give ourselves permission to imagine." 2. "Take a few minutes to think about this - you can close your eyes or take notes on a piece of paper."     Give people 2-3 minutes of head's down time.  Specific prompts to as guides as people close their eyes/take notes   1. How would it begin? What would happen first? 2. What would your role be about? What would your focus be on? 3. What would you do (tasks)? 4. What would you no longer do? 5. Who would be there? 6. Who would you talk to? What would the conversation look like? 7. What supports or systems would be in place to help you? 8. How would it end? 9. How would you feel? 10. About the space? 11. About the people you are interacting with? 12. About your role?     Ask people to share back. Note taker to capture via stickies.    Follow up specifics during the conversation (as needed)   1. What would an interaction with a patient look like, if any? 2. What would an interaction with your supervisor? If any 3. Interaction with a clinician? If any 4. Who might you interact with that you don't interact with today? |
| **Mapping Opportunities**  **(20 min)** | "Let's take a few minutes to think about what are ways in which your experience as an MOA could be improved. We will have more time to ideate together in our next session but we wanted to get started. For example [X suggested Y the other day]".    "So, in what ways could your experience as an MOA be improved? Think about:   1. **People:** Your role, other people's roles, relationships, patients 2. **The Clinic:** How the clinic works, softwares, paperwork, etc. 3. **The System:** Work policies related to MOA's"   Now ask people to share    Note taker to take notes on the circle to visualize the different levels |
| **Next Steps and Close (5 min)** | 1. Thank you 2. Next meeting - we share back a summary of the key opportunities that emerge through our discussions and start ideating concrete solutions. 3. The Miro board is always open - you can add more or edit as you like. 4. We would also love to hear your thoughts and feedback - what worked, what didn't and why - you can add it on the Miro or email us at anytime |

**Meeting 3**

| **Challenges recap + prioritization (10 min)** | Recap of Challenges from meeting 1 + 2   1. Do these challenges summarize or bring to life your experience and what is most challenging as MOA?   Prioritization   1. What do you think is most urgent to address? Which challenges do you think we should focus on solving that would have the biggest impact on your everyday?   Align on 2-3 top challenges |
| --- | --- |
| **Warm-up (5 min)** | Yes, and exercise   1. Ask them to start with the "but" - 2 rounds 2. How far in our trip planning did we get? not very far! 3. Ask them to do the "yes and" - 2 rounds |
| **Opening (5 min)** | "The "yes, and" exercise is a simple exercise to get us in the space for thinking generatively and optimistically. Today is all about thinking of ways we can solve the problems we identified in the past 2 meetings."   - "Here are a few invitations to keep in mind when we ideate" - Share the ideation invitations |
| **Ideation - deep diving into the opp area (15 min)** | "We want to start by diving deeper into the challenge area and mapping what we see as the root causes of the issue. This will help develop more impactful solutions."    Read out the challenge area and ask the group to reflect on what might be causing this problem.   1. Give people space to discuss. 2. Prompt why might this be happening to prompt deeper conversation. |
| **Ideation - open ideation (20 min)** | "Now let's start thinking about ways we might solve this problem! Remember there are no stupid ideas!"   - Restate challenge area and problem and ask "How might we solve this problem, consider: - What types of **supports/resources** would have to be created? - What new **technologies** would need to be available to you? - What new **partnerships or relationships** would need to exist? - What **communications or processes** would need to be designed? - What new **roles** would need to be created? - Give people 3-5 minutes for individual ideation - capture as many ideas as you can - Share back of ideas - note taker captures on board - Invite people to build off each others ideas     Prompts to inspire new thinking   - **Circle back to root causes:** "What would need to be created to solve the root cause X we discussed earlier" - **Share scoping review ideas that were matched:** "Here are some ideas that already exist out there to support MOAs. Do you feel this would help solve the problem? Why or why not? Is this relevant to your practice? What would need to change?" - **What ifs:** Share "what ifs" for inspiration - What could be at the clinic level (how the clinic is run, processes, roles and resp) done to solve this? - What could be done at the community level (outside the clinic, in community) to solve this? - What could be done at a systems level (how the health care system is structured) to solve this? - What would you do if you were in charge? - What if it had no technology involved? - What if money was unlimited? - What if money was limited? - What if you had a lot of time? - What if you had limited time? |
| **Mapping excitements (5 min)** | Give people 2-3 minutes to review ideas and star their top 1-2 ideas: what are you most excited about? |
| **Ideation - refinements on top ideas (20 min)** | Build out top ideas  Prompts from facilitators to help people refine the ideas   1. **What value does this idea provide?** 2. Detail out how the idea would be used 3. **What components (features) would be included in this idea?** 4. How would this idea come to life? 5. What steps took place to ensure this idea was successful 6. Who would need to be involved in developing and implementing this idea to ensure its successful |
| **Next Steps and Close**   **(5 min)** | - Thank you - Next meeting - we work on the second opp space - The Miro board is always open - you can add more or edit as you like. - We would also love to hear your thoughts and feedback - what worked, what didn't and why - you can add it on the Miro or email us at anytime |

**Meeting 4**

| **Check-in (10 min)** | Peach and pit   1. Share something sweet (peach) and something more bitter (pit) about your long weekend |
| --- | --- |
| **Ideation - deep diving into the opp area (15 min)** | "We want to start by diving deeper into the second opportunity area and mapping what we see as the root causes of the issue. This will help develop more impactful solutions."    Read out the opportunity area and ask the group to reflect on what might be causing this problem.   - Give people space to discuss. - Prompt why might this be happening to prompt deeper conversation. |
| **Ideation - open ideation (20 min)** | "Now let's start thinking about ways we might solve this problem! Remember there are no stupid ideas!"   1. Restate challenge area and problem and ask "How might we solve this problem, consider: 2. What types of **supports/resources** would have to be created? 3. What new **technologies** would need to be available to you? 4. What new **partnerships or relationships** would need to exist? 5. What **communications or processes** would need to be designed? 6. What new **roles** would need to be created? 7. Give people 3-5 minutes for individual ideation - capture as many ideas as you can 8. Share back of ideas - note taker captures on board 9. Invite people to build off each others ideas |
| **Ideation - open ideation (20 min)** | Prompts to inspire new thinking   - **Circle back to root causes:** "What would need to be created to solve the root cause X we discussed earlier" - **Share scoping review ideas that were matched:** "Here are some ideas that already exist out there to support MOAs. Do you feel this would help solve the problem? Why or why not? Is this relevant to your practice? What would need to change?" - **What ifs:** Share "what ifs" for inspiration - What could be at the clinic level (how the clinic is run, processes, roles and resp) done to solve this? - What could be done at the community level (outside the clinic, in community) to solve this? - What could be done at a systems level (how the health care system is structured) to solve this? - What would you do if you were in charge? - What if it had no technology involved? - What if money was unlimited? - What if money was limited? - What if you had a lot of time? - What if you had limited time? |
| **Mapping excitements (5 min)** | Give people 2-3 minutes to review ideas and star their top 1-2 ideas: what are you most excited about? |
| **Ideation - refinements on top ideas (25 min)** | Build out top ideas  Prompts from facilitators to help people refine the ideas   1. What value does this idea provide? 2. Detail how the idea would be used 3. What components (features) would be included in this idea? 4. What steps took place to ensure this idea was successful |
| **Next Steps and Close**   **(5 min)** | 1. Thank you 2. What happens next 3. We synthesize all your ideas 4. We will share them back to get more feedback and further prioritize through a survey 5. We will also reach out to hear if you are interested in taking part in any other workshops 6. The Miro board is always open - you can add more or edit as you like. 7. We would also love to hear your thoughts and feedback - what worked, what didn't and why - you can add it on the Miro or email us at anytime |

**Final co-creation workshop**

| **Time** | **Item** | **Activity** |
| --- | --- | --- |
| 10 minutes | Introduction to the project  (large group) | Setting the stage |
| 2 minutes | Arrival | Quick poll on comments  "When thinking about MOAs contribution to primary care, what comes to mind?"    Waterfall technique - Give you 30 seconds to answer, please do not push enter yet - we will do it all at the same time so that we can see people's answers without influence! |
| 5 minutes | Share back of learnings so far  (large group) | Insights story   1. methods 2. key learnings around challenges 3. prioritized solutions (3) 4. explain why we are not working on centralized referral system |
| 10 minutes | Warm up  (small groups) | Ask people to:   1. Where you are located 2. A community or place that is meaningful to you, and why |
| 39 minutes | Solution discussion (small group) | 1. **Discussion on impact (5 min)**: Why did you prioritize this idea?  Facilitators: allow ppl to jump in - if one person is taking up space, for next one, give people 1 min of solo thinking time to take notes on note pad or board 2. **Refine the solution the prioritized (15 minutes)**: 3. Is there anything missing in this idea that would make it even more impactful? 4. Facilitator prompt people to read over the considerations to inspire new thinking 5. **Create a roadmap (15 minutes)** 6. Share an example of roadmap to make sure people are getting specific 7. What would you do next for this idea to successfully come to life in ONE YEAR? 8. Place ideas along the roadmap timeline 9. have prompts on types of things that would be in roadmap 10. Already place the original steps on the roadmap 11. Is there anything you think would get in the way of this happening? 12. Now go back to the roadmap and adapt them to overcome these barriers 13. **Prepare your share back** (4 minutes). Align on one person who will share back 14. Read the title 15. What is most exciting about the solution 16. What are the most important next steps for this to come life? |
| 18 minutes | Discussion  (large group) | 5 minutes per group  1-2 minutes of discussion |
| 5 minutes | Close (large group) | Thank you and next steps    We would love your feedback - please let us know via email or on the board |

**Appendix 2: Co-created solutions developed by Medical Office Assistants**

| **Solution** | **What problem are we solving for?** | **Details about this solution** | **What would need to happen for this to come to life?** |
| --- | --- | --- | --- |
| **Solution 1**  Provincial-wide MOA network: Supporting, advocating for, and advancing the interests of MOAs | **MOAs feel isolated and disconnected from other MOAs**    They have nowhere to turn to for guidance, resources, professional development, and training.    MOAs feel under recognized and undervalued in their role | **The association would support MOAs across provinces. Specifically, it would:**    Advocate for the recognition of MOAs as integral members of healthcare teams and push for improved wages, benefits, and working conditions    Act as a resource with updates on new laws and regulations    Foster a sense of community by providing platforms for networking and sharing best practices    Provide ongoing professional development opportunities, on topics like medical terminology, EHR systems, and patient communication | **Engage MOAs across provinces and identify key issues**    Partner with government, associations, colleges/universities    Figure out sustainable funding |
| **Solution 2**  Patient Navigation Specialist: A new role to simplify health care access | **Patients (especially complex and older patients, newcomers, people with language barriers) often struggle to navigate the healthcare system.**    MOAs want to support patients, but don't have the time and capacity to do so | **Patient Navigation Specialist is available for face-to-face support. They can assist with:**    Act as a point of contact for patients needing extra assistance, addressing questions and concerns (e.g., tech support)    Help patients book appointments, tests, and managing referrals    Connecting patients with community resources and programs | **MOAs would need to be relieved of certain administrative duties or this could be done on a rotation where one MOA takes the role for a day**    Could be piloted with government funding    Would require training |
| **Solution 3** Fostering Care between MOAs and Patients: Training and procedures to support safety and enabling caring relationships | **MOAs feel that they receive the brunt of patients' frustration with the system**    Some patients treat them poorly because of their status within the clinic.    MOAs mental health is severely impacted.    MOAs can feel ill-equipped to address these interactions    MOAs don't always have the support from staff/physicians.    MOAs don't always feel confident in how to approach specific patient communities (e.g., LGBTQ2s+) with care | **Encouraged, subsidized, incentivized training on building caring relationships in clinic. This would include:**    For MOAs: Training on caring relationships with patients - including how to deal with harassment, how to diffuse situations, compassion for patients (e.g., LGBTQ2s+, mental health).    For managers and physicians: Training on how to support MOAs when faced with harassment.  How to develop and implement clear procedures in office on how to respond to challenging encounters | **Create a centralized list of trainings. Ensure this includes trainings that are interactive and engaging.**    Develop trainings where gaps exist.    Incentives/funding for clinics to take trainings |
| **Solution 4** Province-wide Health-Care System Education Campaign: Empowering patients to navigate the system and lower the burden on MOAs | **Lack of understanding among patients on how HC systems works. MOAs feel they have to navigate the system on their behalf.**    Increased burden on MOAs to manage patient expectations that may not align with system capacity | **Province-led training for the general public on navigating the health care system including topics like:**    Understanding the healthcare system    Where and from whom to get help when you have a health-care problem    Making the most of your visit with your provider (e.g.,how to get what you need from an appointment)    Patient rights, roles, and responsibilities (versus what they can expect from the clinic)    How to access community and social support services    How to use digital tools and resources | **This could come to life as:**    Awareness campaign for general public    Series of classes. For example, classes offered in high school (similar to financial literacy classes)    Website with education materials    **This would require:**    Provincial lead to support development of materials or classes    Materials/campaign/classes should be co-created with key stakeholders, including physicians, MOAs, and clinic managers |
| **Solution 5** Clinic-based education initiative: Educating patients on clinic process, roles, and expectations to lower MOA burden and build shared understanding | **Increased burden on MOAs to manage patient expectations that may not align with system capacity.**    Physicians don't always provide consistent information to patients, leaving MOAs to educate (e.g., costs of forms, turnaround times for results or appointments) | **Clinic-based educational initiative to help patients understand clinic processes, roles, and expectations, including:**    Information on MOA roles and expectations    Standard times for referrals, forms, tests, prescription renewal    Outlining of what is billable and what is not under OHIP    How to use digital programmes    Patient, rights. roles, and responsibilities    Explaining how billing works in the clinic to build empathy for physicians and MOAs | **Create a guide that can be shared with patients at onboarding**  **As a regular reminder: in the waiting room, on screens, online portals, etc.**    **This would happen through:**    Provincial bodies could create a template for clinics that could be easily customizable    Identifying staff members who can champion the initiative |
| **Solution 6** Best practices for clinics: Helping physicians manage their clinic and make the most of MOAs | **Doctors are not trained to run a clinic/business, leaving MOAs to figure it out themselves**    MOAs and clinicians scramble to find resources on how to effectively manager the clinic | **Creating a collection of best practices for clinics, to ensure they are making the most out of their MOAs and creating efficient clinic workflows. This document could include:**    Efficient use of technology (e.g., implementing an automated systems for appointment booking, patient reminders, and follow-ups and electronic health records optimization)    Effective communication (e.g., the importance of regular meetings, how to establish efficient communication channels and feedback mechanisms)    Recommendations for patient flow management (e.g., best practices around a triage system, check in and check out procedures)    Time management and workflow automation (e.g., giving MOAs dedicated time to do administrative work)    Ongoing training and development recommendations | **Resource (e.g., website, guidebook, etc.) to be co-created with MOAs, patients, allied health and clinicians from various types of clinics throughout the provinces**    Socialization and implementation of the resource with support from key provincial associations |
| **Solution 7** Maximizing usage of EMRs and digital tools: Training, support and incentives for full adoption | **Clinics don't always use digital platforms to their full potential**    This leads to frustration among MOAs and increased workload  MOAs have to negotiate between different physicians preferences to use or not use certain functions | **Implement training and financial incentives for full adoption of digital tools (such as EMRs):**    Provide comprehensive training for both doctors and MOAs on the platforms and all of their functions.    Introduce financial incentives and funding to support the adoption and use of digital tools to their full capacity. | **Fund, develop, and deliver training programmes**    Possibility to collaborate with digital tool vendors to improve functionality and better integrate the tool within clinic processes    Collaborate with and support existing local intiatives focused on this (e.g., in Ontario—OntarioMD) |
| **Solution 8** Centralized referral system for specialists: Seamless specialist referral and simplified care coordination | **MOAs find the referral process time consuming and hard to navigate. This leads to patients being frustrated and MOAs feeling overwhelmed:**    Specialists currently accept referrals differently - it is a fragmented process (mix of phone calls, faxes, or paper forms)    Lack of transparency around who is accepting referrals, for what problems, wait times, and where the patient is in the referral process | **A platform designed to centralize all referral requests, tracking and communication. The platform would include:**    A comprehensive directory of specialists, including their areas of expertise, availability, wait times, can help primary care clinicians make informed referral decisions.    Facilitate direct scheduling with the specialist’s office, allowing patients to book appointments without back-and-forth coordination.    Provides clear oversight of referral pathways, preventing missed or lost referrals. | **Provincial endorsement and possibly mandating the adoption of the system.**    Funding to develop, implement, and maintain the centralized referral system.    Specialist and primary care clinicians to be involved early to ensure their needs are met.    Collaborate with tech companies specializing in healthcare IT |

**Appendix 3: Delphi Round 1 Results, N=32**

|  | **Overall Score** | **Acceptability** | **Practicality** | **Effectiveness** | **Affordability** | **Side-Effects** | **Equity** |
| --- | --- | --- | --- | --- | --- | --- | --- |
| **Ideas** | **Mean (SD)** | **Mean (SD)** | **Mean (SD)** | **Mean (SD)** | **Mean (SD)** | **Mean (SD)** | **Mean (SD)** |
| **Concept Card 1**  Provincial-wide MOA network: Supporting, advocating for, and advancing the interests of MOAs | 3.68 (0.81) | 3.9 (0.7) | 3.8 (0.8) | 4.3 (0.6) | 3.5 (0.7) | 2.9 (1.1) | 3.7 (0.9) |
| **Concept Card 2**  Patient Navigation Specialist: A new role to simplify health care access | 3.43 (0.95) | 3.7 (1.0) | 3.0 (1.1) | 3.5 (1.0) | 3.8 (0.7) | 2.8 (1.0) | 3.9 (0.8) |
| **Concept Card 3** Fostering Care between MOAs and Patients: Training and procedures to support safety and enabling caring relationships | 4.01 (0.81) | 4.3 (0.7) | 3.7 (1.3) | 4.3 (0.6) | 4.2 (0.6) | 3.2 (0.9) | 4.3 (0.7) |
| **Concept Card 4** Province-wide Health-Care System Education Campaign: Empowering patients to navigate the system and lower the burden on MOAs | 3.29 (0.96) | 3.4 (1.1) | 3.4 (0.9) | 3.2 (1.1) | 3.3 (0.9) | 3.0 (0.8) | 3.3 (0.9) |
| **Concept Card 5** Clinic-based education initiative: Educating patients on clinic process, roles, and expectations to lower MOA burden and build shared understanding | 3.70 (0.96) | 3.7 (1.0) | 3.9 (1.0) | 3.9 (1.0) | 3.8 (1.0) | 2.9 (0.9) | 3.8 (0.9) |
| **Concept Card 6** Best practices for clinics: Helping physicians manage their clinic and make the most of MOAs | 3.67 (0.89) | 3.9 (0.9) | 3.6 (1.0) | 4.3 (0.6) | 3.5 (1.0) | 3.2 (0.9) | 3.6 (1.0) |
| **Concept Card 7** Maximizing usage of EMRs and digital tools: Training, support and incentives for full adoption | 3.72 (0.85) | 4.1 (1.0) | 3.8 (0.9) | 3.9 (0.9) | 3.6 (0.9) | 3.0 (0.8) | 3.8 (0.7) |
| **Concept Card 8** Centralized referral system for specialists: Seamless specialist referral and simplified care coordination | 3.98 (0.99) | 4.3 (1.0) | 4.0 (1.0) | 4.3 (0.9) | 3.9 (1.1) | 3.1 (1.1) | 4.4 (0.8) |

* Average of acceptability, practicality, affordability

| Dark Blue | 4-5 |
| --- | --- |
| Medium Blue | 3.5-3.9 |
| Light Blue | 0-3.4 |

**Appendix 4: Participant Scores for Delphi Round 1 ranked in order, N= 32**

| **Solution** | **MOAs**      **N= 12** | **Physicians**      **N= 9** | **Health Systems Leaders**  **N= 11** | **Overall**      **N= 32** |
| --- | --- | --- | --- | --- |
| Fostering Care between MOAs and Patients | 4.5 | 4.1 | 3.7 | 4.1 |
| Centralized referral system for specialists | 4.3 | 4.4 | 3.5 | 4.1 |
| Provincial Wide MOA Network | 4.1 | 3.6 | 3.6 | 3.8 |
| Maximizing usage of EMRs and digital tools | 4.0 | 3.9 | 3.5 | 3.8 |
| Clinic-based education initiative | 4.2 | 3.7 | 3.3 | 3.7 |
| Best practices for clinics | 4.1 | 3.7 | 3.4 | 3.7 |
| Patient Navigation Specialist | 4.2 | 3.3 | 3.2 | 3.6 |
| Province-wide Health-Care System Education Campaign | 3.8 | 3.3 | 3.3 | 3.5 |

| Purple | MOAs highest scores |
| --- | --- |
| Green | Physicians Highest Scores |
| Blue | Health Systems Leaders Highest Scores |
| Red | Participants lowest scores |

**Appendix 5: Survey 2 Results: Mean ranking Solutions by Effectiveness, Feasibility, Side Effects, Equity, and Priority**

| **Solution** | **Mean ranking: Effectiveness** | **Mean ranking: Feasibility** | **Mean ranking: Side Effects** | **Mean ranking: Equity** | **Mean ranking: Highest Priority** |
| --- | --- | --- | --- | --- | --- |
| Fostering Care between MOAs and Patients | 4.3 | 2.5* | 2.8* | 3.1 | 2.8* |
| Centralized referral system for specialists | 3.95 | 4.4 | 3.7 | 2.4* | 3.1 |
| Provincial Wide MOA Network | 2.4* | 3.7 | 6.2 | 4.7 | 2.8* |
| Maximizing usage of EMRs and digital tools | 3.7 | 4.4 | 4.1 | 4.9 | 4.6 |
| Clinic-based education initiative | 5.5 | 4.1 | 4.9 | 5.1 | 5.5 |
| Best practices for clinics | 4.4 | 5.4 | 4.6 | 5.6 | 4.1 |
| Patient Navigation Specialist | 5.4 | 5.3 | 5.8 | 3.9 | 5.8 |
| Province-wide Health-Care System Education Campaign | 6.35 | 6.4 | 3.8 | 6.3 | 7.3 |

*Highest ranking in that category

**Appendix 6: Final Co-created solutions after the final workshops**

| **Patient Access to EMRs: Empowering patients with EMR access to reduce clinic workloads** | | |
| --- | --- | --- |
| **Problem** | **Solution** | **How** |
| - MOAs feel overwhelmed by heavy workloads - part of this includes managing phone calls to retrieve information from the EMR for patients - Wait times at the clinic are long because of the high level of demands MOAs are managing - Patients feel that they don't have control over their health records and often feel as though doctors act as gatekeepers | Grant patients access to their Electronic Medical Records (EMR) through a user-friendly patient portal, enabling them to view their health information and reduce clinic workload. This could include:   - Main documents, instructions, test results, wait times for referrals, and appointment history - Could also include resources for patients, educational materials, administrative policies, and community resources. | This could happen in different ways:   - Supporting and working with local intiatives that are already trying to centralize patient-facing EMRs such as in Ontario "Connecting Ontario" (initiative through Ontario Health to enable patients to access EMR records) - Working with EMR vendors to design patient versions within existing EMRs (similar to what already exists within EPIC) |

| **Maximizing the EMR and digital tools: Training, Support, and Incentives for Full Adoption** | | |
| --- | --- | --- |
| **Problem** | **Solution** | **How** |
| - Clinics don't always use digital platforms to their full potential - This leads to frustration among MOAs and increased workload - MOAs have to negotiate between different physicians preferences to use or not use certain functions | Implement training and financial incentives for full adoption of digital tools (such as Ocean, EMRs, etc.):   - Provide comprehensive training for both doctors and MOAs on the platforms and all of their functions. - Introduce financial incentives and funding to support clinics and providers in adopting and using digital tools to their full capacity. - Ensure there are clinic processes in place to enable this. For example, ensuring that email addresses are consistently collected and updated for all patients. - Possibility to collaborate with digital tool vendors to improve functionality and better integrate the tool within clinic processes | - Secure financial support for clinics to cover the costs of implementation, training, and ongoing usage of digital tools - Develop and deliver training programmes to ensure proficiency with tools. - Collaborate with digital tool vendors to improve functionality - Collaborate with and support existing local initiatives focused on this (e.g., in Ontario—OntarioMD) |

| **Centralized referral system for specialists: Seamless specialist referral and simplified care coordination** | | |
| --- | --- | --- |
| **Problem** | **Solution** | **How** |
| MOAs find the referral process time consuming and hard to navigate. This leads to patients being frustrated and MOAs feeling overwhelmed:   - Specialists currently accept referrals differently - Lack of transparency who is accepting referrals, for what problems, wait times - Lack of transparency and communication regarding where patient is in the referral process (i.e., triage, booked, a consultation note ready) | A streamlined platform designed to manage and coordinate patient referrals from primary care providers to specialists. Instead of relying on fragmented processes—such as phone calls, faxes, or paper forms—the system centralizes all referral requests, tracking, and communication within a single digital interface. The platform would include:   - A comprehensive directory of specialists, including their areas of expertise, availability, wait times, can help primary care providers make informed referral decisions. - Facilitate direct scheduling with the specialist’s office, allowing patients to book appointments without back-and-forth coordination. - All referrals would go through this system - Provides clear oversight of referral pathways, preventing missed   or lost referrals. | - **Provincial Endorsement**: The Ontario Ministry of Health would need to endorse the initiative and possibly mandate the adoption of the system. - **Funding Allocation**: resources to develop, implement, and maintain the centralized referral system. - **Specialist and primary care providers** to be involved early to ensure their needs are met. - Collaborate with **tech companies** specializing in healthcare IT |

| **Patient Navigation Specialist: A new role to simplify healthcare access** | | |
| --- | --- | --- |
| **Problem** | **Solution** | **How** |
| - Patients often struggle to navigate the healthcare system. - MOAs want to support patients, but don't have the time and capacity to do so - Older patients usually need more support, especially for complex appointments where they have to see multiple specialists. - Language barriers can make navigation even harder for patients | Positioned in the clinic, the Patient Navigation Specialist is available for face-to-face support. This role is critical in big clinics where there are multiple doctors and a lot of patients. This could be a new role or MOAs could establish a rotation where one of them takes on that role for the day. They can assist with:   - Tech support - Help patients book their own appointments - Manage onboarding processes, ensure all necessary forms are completed accurately - Act as a point of contact for patients, addressing questions and concerns | - MOAs would need to be relieved of certain administrative duties or this would have to be a standalone role - Physicians and management would need to create this new role, along with MOA expertise. - Could be piloted with additional government funding - Would require a training program for the navigation specialist - Would require educating patients on the availability of this support role |

| **MOAs as Health Coaches: A new role to support and motivate patients in lifestyle changes** | | |
| --- | --- | --- |
| **Problem** | **Solution** | **How** |
| - MOAs' heavy workload means they often feel like they cannot properly meet patient needs, especially those who need more care or time - Patient care is what attracted MOAs to the role in the first place - but they often feel they don't have time to do it - RPNs may be overwhelmed by workload and may not have the time to coach patients as needed | MOAs often wish they were more involved in patient care. This dedicated health coach would support patients in life style changes, freeing up time for RPNs and enabling MOAs to take on a more active role in patient care. This role could rotate between MOAs or be held by a dedicated person. The health coach would support patients in:   - Assessment of current lifestyle (e.g., smoking cessation) - Ensure screening is up to date (e.g., cancer, vaccinations) - Health education on topics like nutrition and exercise - Skill development for diet, exercise, sleep etc. - Connecting patients with community resources and programs | - MOAs would need to be relieved of certain administrative duties or this would have to be a standalone role - Physicians and management would need to create this new role, along with MOA expertise. - Could be piloted with additional government funding - Would require training for MOAs - Would require building a process/workflow that enables physicians to give directives to MOAs and ensure coverage under physician insurance - Would require educating patients on the availability of this support role |

| **Health-care System Navigation Education: Empowering Ontarians to navigate the system and lower the burden on MOAs** | | |
| --- | --- | --- |
| **Problem** | **Solution** | **How** |
| - Many people are unaware of how to navigate health care system - The burden falls on MOAs to navigate the system on their behalf - There is an expectation that MOAs should complete tasks for patients, who have capacity to do so themselves - especially found amongst the younger generation e.g. appointment reminders | Training the general public on navigating the health care system. This can be offered in high school (similar to financial literacy classes), as well as to newcomers who have not experienced the Ontario health care system yet. This could be done through a series of classes, an awareness campaign, or hosted on a portal. Educational material for the general public can also be created and shared on common tasks such as booking appointments. This educational initiative could include:   - Understanding Ontario's Healthcare System - How to access care at the right place and the right time - Understanding appointments and wait times - Making the most of your visit with your provider - Patient rights - Patient roles and responsibilities in managing their health (versus what they can expect from the clinic) - How to access community and social support services - How to use digital tools and resources (e.g. My Chart) | - Provincial Lead: to support development of educational materials and promotion to public - Educational materials should be co-created with key stakeholders, including physicians, MOAs, and clinic managers |

| **Clinic Clarity: Educating patients on clinic process, roles, and expectations to lower MOA burden and build shared understanding** | | |
| --- | --- | --- |
| **Problem** | **Solution** | **How** |
| - Patient expectations for appointments and turnaround times do not align with how the clinic/system works - Lack of understanding among patients on how HC systems works and typical referrals times - Increased burden on MOAs to manage expectations - Physicians don't always provide consistent information to patients, leaving MOAs to educate (ex: costs of forms, turnaround times for results or appointments) | Clinic-based educational initiative to help patients understand clinic processes, roles, and expectations. This could be shared with patients when being onboarded, but would also need to be shared as regular reminders to patients (e.g., in the waiting room, on screens, online portals, etc.). This could include:   - Information on MOA roles and expectations - Standard times for referrals, forms, tests, prescription renewal - Outlining of what is billable and what is not under OHIP - How to use digital programmes (e.g., Ocean) - Patient roles and responsibilities - Explaining how billing works in the clinic to build empathy for physicians and MOAs | - Support from clinic management and lead physicians - Resource assessment to evaluate budget for materials and staff time - Identify staff members who can champion the initiative - Provincial bodies could create a template for clinics that could be easily customizable - Engage and co-create with patients, MOAs, and physicians |

| **MOAs are ESSENTIAL to primary care teams: Best practices on making the most out of MOAs in primary care clinics** | | |
| --- | --- | --- |
| **Problem** | **Solution** | **How** |
| - Doctors are not trained to run a clinic/business, leaving MOAs to figure it out themselves. - Physicians often focus on medical care, leaving MOAs to manage the running of the clinic, with little to no time/resources (e.g. technological tools, referral process, communication within the clinic team) - MOAs have to go looking for resources and there is no one to guide them | Creating a collection of best practices for clinics, to ensure they are making the most out of their MOAs and creating efficient clinic workflows. This document could include:   - Efficient use of technology (e.g., automated systems for appointment booking, patient reminders, and follow-ups and Electronic Health Records Optimization) - Effective Communication (e.g., regular meetings, efficient communication channels and feedback mechanisms) - Recommendations for patient flow management (e.g., triage system, check in and check out procedures) - Time Management and Workflow Automation (e.g., time blocking and batch processing) - Ongoing training and development recommendations | - Resource to be co-created with MOAs, patients, allied health and clinicians from various types of clinics throughout the provinces - Support from key provincial associations - Encouragement and incentives to engage and implement the resource |

| **Fostering care between MOAs and Patients: Training and procedures to support safety and enabling caring relationships** | | |
| --- | --- | --- |
| **Problem** | **Solution** | **How** |
| - MOAs feel that they receive the brunt of patients' frustration with the system and that some patients treat them poorly because of their status within the clinic. MOAs mental health is severely impacted. - MOAs can feel ill-equipped to address these interactions, clinics don't always have an agreed-upon standards on how to respond, and MOAs don't always have the support from staff/physicians. - MOAs don't always feel confident in how to approach specific patient communities (e.g., LGBTQ2s+) with care | Encouraged, subsidized, incentivized training for MOAs on facilitating caring relationships with patients - including how to deal with harassment, how to diffuse situations, compassion for patients with various experiences and communities (e.g. LGBTQ2s+, mental health). This training would be supported by:   - Clear procedures in office on how to respond to challenging encounters, including guidance on when and how managers and physicians should step in - Centralized list of existing trainings on these topics for MOAs with clear guidance on how to access and funding available. | - Identify existing trainings on these topics and create a centralized list for clinics to access. Develop trainings where gaps exist - Incentives/funding for clinics to take trainings - Provincial support to develop in-clinic procedures on staff safety. - Training for managers and physicians on how to support MOAs when faced with harassment. |

| **Provincial-wide MOA network: Supporting, advocating for, and advancing the interests of MOAs** | | |
| --- | --- | --- |
| **Problem** | **Solution** | **How** |
| - MOAs feel isolated and disconnected from other MOAs - They have no where to turn to for guidance, resources, professional development, and training. - MOAs face challenges with navigating technological and administrative changes, and have little support to help them adapt - MOAs feel under recognized and under valued in their role | The association would support professional development, improve working conditions, advocate for recognition of MOAs, and ensure consistent standards across the province. Specifically:   - Advocate for the recognition of MOAs as integral members of healthcare teams and push for improved wages, benefits, and working conditions. - Act as a resource for MOAs with updates on new laws and regulations - Foster a sense of community among MOAs by providing platforms for networking and sharing best practices. - Advocate for MOAs within healthcare policy discussions, ensuring that their voices are heard in decisions that affect their work environment and role within the healthcare system. - Provide ongoing professional development opportunities, including workshops, webinars, and conferences on topics like medical terminology, EHR systems, and patient communication. | - Engage MOAs across the province and identify key issues - Partner with unions and associations - Figure out sustainable funding - Engage with colleges and universities offering MOA programs to build partnerships - Work with the Ontario Ministry of Health |
